# Proteomic analysis identifies pathways related to immune dysregulation in patients with hematologic malignancies after COVID-19 infection

**DOI:** 10.1128/spectrum.03996-25

**Published:** 2026-07-15

**Authors:** Bo Tang, Xiaodi Yang, Wenmin Tian, Jialin Zhu, Wei Liu, Mingyi Di, Huihui Liu, Zeyin Liang, Yang Chen, Yujun Dong

**Affiliations:** 1 Department of Hematology, Peking University First Hospital 26447, Beijing, China; 2 Center for Precision Medicine Multi-Omics Research, Institute of Advanced Clinical Medicine, Peking University 12465, Beijing, China; 3 Department of Biochemistry and Biophysics, Center for Precision Medicine Multi-Omics Research, School of Basic Medical Sciences, Peking University Health Science Center 33133, Beijing, China; University of Manitoba, Winnipeg, Manitoba, Canada; Universidade Federal de Uberlandia, Uberlandia, Brazil

**Keywords:** COVID-19, hematologic malignancies, immune dysregulation, platelet activation, myocardial dysfunction

## Abstract

Patients with hematologic malignancies (HMs) are particularly vulnerable to coronavirus disease 2019 (COVID-19) because of underlying immune dysfunction and treatment-related immunosuppression. However, proteomic features associated with different clinical trajectories in this population remain insufficiently characterized. We performed serum proteomic analysis in 40 HM patients with COVID-19 and 15 healthy controls. Compared with controls, HM patients showed impaired immune-related responses during the acute phase of COVID-19. Acute-phase proteomic patterns differed across outcome groups; however, because outcome groups were closely intertwined with initial COVID-19 severity, ICU admission, and systemic illness, and because multivariable adjustment was not performed due to the limited sample size, these patterns should be interpreted as severity- and outcome-associated profiles rather than independent trajectory-specific markers. Fatal cases showed evidence of dysregulated immune activation, whereas patients later classified as having long COVID exhibited broader suppression of immune-related pathways. In addition to immune alterations, pathways related to platelet activation and cardiac-related dysfunction were associated with adverse clinical trajectories. Enzyme-linked immunosorbent assay validation supported the association of selected proteins with outcome groups during acute infection. These findings provide a proteomic overview of COVID-19 in HM patients and offer a basis for future mechanistic studies and larger external validation cohorts.

**IMPORTANCE:**

Patients with hematologic malignancies are highly vulnerable to severe coronavirus disease 2019 (COVID-19), acute death, and long COVID due to preexisting immune dysfunction. However, the proteomic signatures linked to adverse clinical trajectories remain poorly understood. Our serum proteomic study identifies distinct acute-phase immune profiles associated with different outcomes: broad immune suppression characterizes long COVID, while dysregulated immune activation is associated with fatal cases. Platelet activation and cardiac-related pathways are also linked to poor outcomes. These findings provide key molecular insights for this high-risk population, supporting future biomarker development, risk stratification, and targeted clinical management.

**CLINICAL TRIALS:**

This study is registered with ClinicalTrials.gov as NCT05683353.

## INTRODUCTION

Coronavirus disease 2019 (COVID-19), caused by the severe acute respiratory syndrome coronavirus 2 (SARS-CoV-2), was declared a pandemic by the World Health Organization (WHO) in March 2020 (1). Since then, the global spread of COVID-19 has resulted in over 1.5 million deaths worldwide (2), with clinical manifestations ranging from mild fever to severe lung injury (3).

Patients with hematologic malignancies (HMs) face heightened vulnerability to severe infections due to malignancy-associated immune compromise and frequent immunosuppressive therapies (4). This population experiences elevated risks of life-threatening complications and is more likely to be affected by persistent post-acute symptoms, clinically defined by the WHO as “long COVID,” which can exert lasting health impacts (5, 6). Given the potentially fatal and debilitating nature of COVID-19 in HM patients, this population warrants focused attention. COVID-19’s impact on multiple organ systems and diverse complications make it a critical area of concern for HM patients for both clinical management and long-term care (7–10).

Mass spectrometry (MS)-based proteomics provides a powerful platform for comprehensive proteomic analysis in complex biological samples, such as plasma or serum (11, 12). This technique enables the unbiased identification and quantification of a broad spectrum of proteins, which is essential for discovering novel biomarkers and understanding disease mechanisms. Importantly, proteomic analysis can reveal underlying disorders before they are detectable by conventional clinical indicators.

In this study, we recruited a cohort of 40 HM patients during acute-phase COVID-19 infection. We performed quantitative proteomic analysis on patient serum samples and integrated these data with clinical results from routine blood tests. Our analysis revealed compromised immune responses in HM patients during acute COVID-19 compared to healthy controls. Notably, immune profiles diverged significantly according to clinical outcomes: patients progressing to long COVID demonstrated more pronounced immune suppression, while fatal cases displayed signs of abnormal immune activation during the acute phase. Beyond immune dysregulation, we identified platelet activation and myocardial dysfunction as key determinants of clinical outcomes. Enzyme-linked immunosorbent assay (ELISA) validation further supported the association of selected acute-phase proteins with clinical outcome groups in this vulnerable population.

This present study focuses on a clinically significant yet understudied population: patients with HMs. Given that these patients exhibit baseline immune dysregulation and treatment-related perturbations, such factors may alter the proteomic signatures associated with COVID-19 infection. We explicitly illustrate that this study integrates stage-specific sampling with orthogonal ELISA validation to ensure the reliability and robustness of our findings. Specifically, we highlight three principal novel findings: (i) distinct acute-phase immune patterns that correlate with subsequent clinical trajectories, characterized by more pronounced immune suppression in patients who later developed long COVID and immune hyperactivation in fatal cases; (ii) perturbations in platelet activation- and cardiac-related pathways that are associated with adverse clinical outcomes; and (iii) ELISA validation of a targeted protein panel (ARRB1, CCL5, FLT3, BTK, ITGA2B [integrin alpha-IIb], ITGB3 [integrin beta-3]) that is closely linked to outcome groups. Our study aims to elucidate the molecular dynamics of COVID-19 in HM patients, thereby providing deeper insights into the underlying disease pathogenesis. These findings hold significant potential to inform clinical decision-making processes, ultimately contributing to improved prognoses and therapeutic outcomes for this high-risk patient cohort.

## MATERIALS AND METHODS

### Study design and participants

This single-center, prospective study was conducted at Peking University First Hospital, Beijing, China. We enrolled 40 HM patients with laboratory-confirmed COVID-19, together with 15 non-tumor control individuals. Cohort selection and outcome-group assignment are summarized in Fig. S1. A total of 98 peripheral whole blood samples were collected across the study period. For stage-based analyses, samples were categorized as baseline/control (*N* = 16), acute infection (*N* = 48), and post-infection convalescence (*N* = 34) stages among hematologic tumor patients and non-tumor individuals according to the timing of SARS-CoV-2 infection and clinical follow-up.

For outcome-based analyses among HM patients, participants were classified into uncomplicated infection (*N* = 25), long COVID (*N* = 7), and death (*N* = 8) groups according to clinical trajectory. Uncomplicated infection is defined as cases in which patients recover completely without developing persistent post-acute symptoms. As for the definition of long COVID, we complied with the United Kingdom National Institute for Health and Care Excellence guidelines that defined long COVID as persistence of symptoms beyond 4 weeks of SARS-CoV-2 infection and considered acute death as patients who deceased within 2 weeks post-infection. Persistent viral positivity was not required for long-COVID classification. We also clarified that participants without adequate follow-up beyond 4 weeks were not assigned to the long-COVID group and were excluded from long-COVID classification analyses. The median interval from SARS-CoV-2 diagnosis to acute-phase blood collection was 1 day (interquartile range, 1–1 days). Overall, 34 patients contributed paired acute- and convalescent-phase samples, and 6 patients contributed a single time-point sample.

Inclusion criteria were age ≥18 years, diagnosis of a hematologic malignancy, and laboratory-confirmed SARS-CoV-2 infection. COVID-19 severity was classified according to NIH guidance (13).

### Depletion of highly abundant proteins from serum samples

High-Select Top14 Abundant Protein Depletion of serum samples was performed following the manufacturer’s protocol for the “Multi Affinity Removal Column, Human-14” (Agilent Technologies). In brief, 20 μL of serum was mixed with 60 μL of buffer A and transferred into a Spin-X centrifuge tube filter (0.22 μm cellulose acetate, COSTOR). The mixture was then centrifuged at 16,000 × *g* for 1 min. The resulting filtrate was collected and transferred into preassembled plastic springs (29 × 5 mm) for High-Select Top14 Abundant Protein Depletion, using an Agilent 1290 liquid chromatography system. The depleted samples were subsequently collected, and protein concentrations were determined using the BCA Protein Assay Kit (Thermo Scientific), according to the manufacturer’s guidelines.

### Sample preparation

A 50 μg aliquot of the depleted serum sample was precipitated by adding one-third of its volume of trichloroacetic acid solution and incubating the mixture overnight at 4°C. The sample was then centrifuged at 16,000 × g for 30 min at 4°C. The resulting precipitate was washed three times with acetone and dried using a vacuum concentrator (Labconco, USA). The dried precipitate was subsequently dissolved in 40 μL of 8 M urea (pH 8.5), treated with 20 mM (2-carboxyethyl) phosphine hydrochloride (TCEP) for reduction, and alkylated with 40 mM iodoacetamide. After incubation, the mixture was diluted with 200 μL of 100 mM Tris-HCl buffer (pH 8.5), achieving a final urea concentration of 1.3 M, and then digested with 3 μg of trypsin at 37°C for 16 h. The digestion products were desalted using a Monospin C18 column (GL Sciences, Tokyo, Japan). The desalted peptides were vacuum-centrifuged to dryness, then reconstituted in Milli-Q water containing 0.1% formic acid (FA) for liquid chromatography-mass spectrometry (LC-MS) analysis. Indexed retention time (iRT) calibration peptides were added to the samples prior to data-independent acquisition (DIA) analysis.

### Sample preparation for spectral library generation

Approximately 70 μg of the mixed peptide sample for spectral library construction was fractionated using a high-pH reversed-phase HPLC system (1290 Infinity, Agilent). Briefly, the mixed peptides were resolved in Buffer A (20 mM ammonium, 2% acetonitrile [ACN], pH 10.5) and loaded onto an Accucore C18 column (2.1 mm × 150 mm, 2.6 μm particles; Thermo Fisher Scientific). A 56 min linear gradient of 5%–80% Buffer B (20 mM ammonium, 98% ACN, pH 10.5) was applied at a flow rate of 0.4 mL/min. Sixty fractions were collected during the liquid chromatography separation and then pooled into 20 fractions by combining fractions 1, 21, 41, and so on. The pooled fractions were dried completely using a vacuum concentrator. The dried peptides were then reconstituted in 10 μL of Milli-Q water containing 0.1% FA and spiked with iRT peptides to a final concentration of 1× for chromatographic calibration.

### LC-MS/MS analysis

The nLC-MS/MS analysis was performed on an EASY-nLC 1200 HPLC (Thermo Scientific) coupled to a Q Exactive HF-X (Thermo Scientific) mass spectrometer. All samples were separated within 90 min on a reversed-phase C18 column (25 cm × 75 µm, 1.7 μm, IonOpticks). The elution gradient of B was linearly increased from 9% to 27% within 70 min, then increased to 45% within 8 min, increase to 95% within 5 min, and continued for another 7 min at a flow rate of 300 nL/min at 50°C (mobile phases A: 0.1% formic acid in water; mobile phases B: 0.1% formic acid in 80% acetonitrile). The mass spectrometer was operated in positive mode using either data-dependent acquisition (DDA) or DIA modes.

### DDA mode

The samples were subjected to analysis using a top 20 DDA approach with the following experimental conditions: Full MS1 scans were conducted in the m/z range of 350–1,200, with a resolution of 60,000, a target AGC value of 3 × 10⁶, and a maximum ion injection time of 50 ms. For MS2 scans, the resolution was set to 15,000, covering the m/z range of 200–2,000, with a target AGC value of 5 × 10⁵ and a maximum injection duration of 120 ms. Ion fragmentation was induced with a normalized collision energy (NCE) of 30, an isolation window of 1.6 m/z, an ion selection threshold of 6.7 × 10⁴, and dynamic exclusion set for 30 s. As a standard quality control, 400 ng of HeLa protein digest (Pierce) was analyzed routinely.

### DIA mode

For single-shot proteomics using DIA, the MS1 scan was performed with a resolution of 60,000, covering a mass-to-charge ratio (m/z) range of 350–1,200. The target AGC value was set to 3 × 10⁶, and the maximum ion injection time was 50 ms. Precursor ions were isolated with a dynamic isolation window spanning from 350 to 1,200 m/z. These precursors were then fragmented via high-energy collisional dissociation with an NCE of 28. MS2 data were acquired using the Orbitrap with a resolution of 30,000. The maximum injection time for MS2 was set to auto, and the AGC target was adjusted to 1 × 10⁶.

### Generation of spectral libraries and DIA data analysis

High-confidence MS/MS spectral libraries were constructed from DDA raw files using the “Generate Spectral Library” module within the Library workflow of Spectronaut (v.16.0, Biognosys). Subsequent DIA data analysis was conducted using the “Set Up a DIA Analysis” module in the Analysis workflow of the same software. Both library generation and DIA analysis employed identical parameter sets, differing only in their respective processing modules. The acquired MS/MS spectra were matched against the human UniProt database (comprising 20,421 human protein entries, downloaded in July 2019). All search parameters were set to their default configurations. DIA data were processed using the default mode. In brief, the search employed carbamidomethylation as a fixed modification, along with acetylation at the protein N-terminus and oxidation of methionine residues as variable modifications. The mass tolerance for precursor ions was set to 10 ppm, and fragment ions to 20 ppm. The Trypsin/P cleavage rule was applied, allowing for up to two missed cleavages. Peptides were required to be at least seven amino acids long, with a maximum length of 52 amino acids. Both the false discovery rate (FDR) thresholds for peptide-spectrum match and protein quantification were set to 0.01.

### Differential expression analysis

Proteomic intensity data were preprocessed using OmicsBean. Missing values were imputed using the DBB algorithm, followed by quantile normalization. Differential expression between comparison groups was assessed using log2 fold-change estimation together with two-sided statistical testing. Resulting *P*-values were adjusted for multiple testing using the Benjamini-Hochberg procedure. Unless otherwise specified, proteins with an absolute log2 fold change >1 and an FDR-adjusted *P*-value (q-value) <0.05 were considered differentially expressed. Ranked differential protein results, including the direction of change, fold change, nominal *P*-values, and adjusted q-values, are provided in the Table S1.

### Enzyme-linked immunosorbent assay

Selected proteins identified in the discovery proteomic analysis were further evaluated by ELISA according to the manufacturers’ instructions. Serum samples were assayed in technical replicates, and sample identity was blinded to the operator during measurement. Absorbance was read at 450 nm, and concentrations were calculated on the basis of the corresponding standard curves. Assay reproducibility was assessed using the intra-assay coefficient of variation. ELISA results were interpreted as orthogonal validation of outcome-associated proteins rather than as evidence of predictive performance.

### Network analysis and drug repositioning

Experimentally validated human protein-protein interactions were obtained from the BioGRID database. After removal of duplicate entries and non-physical associations, interactions involving differentially expressed proteins were extracted to construct an outcome-related interaction network. Functional modules were identified using the MCODE plugin in Cytoscape (include loops = no; degree cutoff = 2; haircut = no; fluff = no; node score cutoff = 0.4; k-core = 2; max depth = 100). KEGG pathway enrichment analysis was then performed for each module using a hypergeometric test with Benjamini-Hochberg-adjusted *P*-values. Drug-target and drug-disease information was obtained from the Therapeutic Target Database (TTD) and mapped to the identified network modules. These analyses were intended to generate biological hypotheses and candidate targets for future study rather than to establish therapeutic recommendations.

### Statistical analysis

Categorical variables are presented as frequencies and percentages, and continuous variables as medians with interquartile ranges unless otherwise specified. Continuous variables were compared between groups using the independent-samples *t*-test (for normally distributed data) or the Mann-Whitney *U*-test (for non-normally distributed data). Categorical variables were analyzed using the χ^2^ test or Fisher’s exact test, as appropriate.

For proteomic analyses, the principal comparisons were conducted as stage-matched between-group analyses. To avoid pseudo-replication, only one sample per patient per stage was included in each comparison. Although paired acute- and convalescent-phase samples were available for a subset of participants, the present study was not designed as a formal within-patient longitudinal mixed-effects analysis, and repeated-measures modeling was therefore not used for the main outcome-group comparisons.

Differential protein analyses were based on effect size estimation together with control of the FDR, and adjusted q-values are reported in the Table S1. Given the modest sample size and the limited number of events in some subgroups, analyses adjusting for potential confounding factors were intentionally parsimonious and are interpreted cautiously. Accordingly, the present study emphasizes descriptive transparency and association-based interpretation rather than definitive causal inference. Heatmaps and network visualizations were generated using ggplot2 and Cytoscape.

## RESULTS

### Clinical characteristics and association with outcomes in HM patients with COVID-19

This study included 40 patients with HMs diagnosed with COVID-19 at Peking University First Hospital. The demographic and clinical characteristics of the cohort are summarized in Table 1. The cohort had a median age of 57.5 years (IQR, 37.25–68.25; range 21-78), comprising 21 males (52.5%) and 19 females (47.5%), with no significant gender-based differences in characteristics. The largest diagnostic subgroup was acute leukemia (*n* = 17, 42.5%), followed by lymphoma (*n* = 10, 25.0%). Comorbidities, including hypertension, diabetes mellitus, and renal impairment, were present in 19 patients (47.5%) (Table 1). At the time of COVID-19 diagnosis, 29 patients (72.5%) had stable underlying disease.

**TABLE 1:** Demographic and clinical characteristics of enrolled patients at COVID-19 diagnosis

	*N*	%
Gender		
Male	21	52.5
Female	19	47.5
Age, median (IQR, range)	57.5 (37.25–68.25), (21–78)	
Comorbidities		
Hypertension	12	30.0
Diabetes	7	17.5
Renal impairment	2	5.0
Diagnosis		
Acute leukemia	17	42.5
Lymphoma	10	25.0
Myelodysplastic syndromes	3	7.5
Multiple myeloma	6	15.0
Others	4	10.0
Status		
Onset	4	10.0
Control	4	10.0
Stable disease	29	72.5
Refractory/Resistant	3	7.5
Vaccination		
Yes	20	50.0
No	19	47.5
NA	1	2.5
Summary of received treatment Chemotherapy		
In the last month	13	32.5
In the last 3 months	9	22.5
Treatment ended >3 months	16	40.0
Chemotherapy		
Yes	13	32.5
No	27	67.5
CD20-targeted therapy		
Yes	7	17.5
No	33	82.5
Anti-infective therapy		
Yes	23	57.5
No	17	42.5

Among the 40 HM patients, 8 died during the acute course of COVID-19, and 32 survived. Among the survivors with adequate follow-up, 7 were classified as long COVID and 25 as the non-long-COVID survivor group. Because of the small subgroup sizes, all outcome-based comparisons were interpreted cautiously.

### Association of clinical characteristics with mortality

Among the 40 HM patients, 8 died during the acute phase of COVID-19, and 32 survived. We analyzed the clinical characteristics and laboratory test results of these patients during the acute phase of infection. No significant differences in age or sex were observed between the two groups in this cohort. Compared with survivors, fatal cases more frequently had severe/critical disease and ICU admission and more often received anti-COVID treatment.

Acute-phase laboratory testing showed significantly lower albumin (ALB) levels (*P* = 0.01) and higher inflammatory markers, including interleukin-6 (IL-6; *P* = 0.031) and procalcitonin (PCT; *P* < 0.001), in the fatal group. In addition, fatal cases showed evidence of coagulation abnormalities, including prolonged prothrombin time (PT) and higher INR values compared with the surviving group (Table 2; *P* = 0.027). These findings are consistent with previous studies, which have shown that COVID-19 patients who succumb to the disease often display heightened inflammatory responses and coagulation dysfunction (14–16).

**TABLE 2:** Clinical features and laboratory tests of HM patients with different outcomes at COVID-19 diagnosis

	Died (*N* = 8)	Survived (*N* = 32)	*P*-value
	*N* (%)	*N* (%)	
Gender			0.241
Male	6 (75.0%)	15 (46.9%)	
Female	2 (25.0%)	17 (53.1%)	
Age, median (IQR, range)	68.5 (36.0–76.5, 23–78)	56.0 (37.25–64.5, 21–74)	0.238
Diagnosis			0.967
Acute leukemia	3 (37.5%)	14 (43.8%)	
Lymphoma	2 (25.0%)	8 (25.0%)	
Myelodysplastic syndromes	1 (12.5%)	2 (6.3%)	
Multiple myeloma	1 (12.5%)	5 (15.6%)	
Others	1 (12.5%)	3 (9.4%)	
Stable disease status			1.000
Yes	6 (75.0%)	24 (75.0%)	
No	2 (25.0%)	8 (25.0%)	
Vaccination status			0.011
Vaccinated	1 (12.5%)	19 (59.4%)	
Unvaccinated	6 (75.0%)	13 (40.6%)	
NA	1 (12.5%)	0 (0.0%)	
Recent chemotherapy			0.236
Yes	1 (12.5%)	12 (37.5%)	
No	7 (87.5%)	20 (62.5%)	
CD20-directed therapy			0.611
Yes	2 (25.0%)	5 (15.6%)	
No	6 (75.0%)	27 (84.4%)	
Antiviral therapy			0.107
Yes	7 (87.5%)	16 (50.0%)	
No	1 (12.5%)	16 (50.0%)	
COVID-19 infection			<0.001
Critical/severe	8 (100.0%)	5 (15.6%)	
Non-critical/severe	0 (0%）	27 (84.4%）	
Admitted to ICU	6 (75.0%）	2 (6.3%）	<0.001
Anti-COVID-19 drugs	7 (87.5%）	16 (50.0%)	0.107
Long COVID-19	0 (0.0%)	7 (21.9%)	0.309
Neutrophils level, median (IQR)	3.30 (0.80–4.39)	3.35 (0.875–4.39)	0.654
Lymphocytes level, median (IQR)	0.43 (0.30–0.80)	0.60 (0.20–0.715)	0.293
PLT level, median (IQR)	51.00 (36.0–166.0)	89.50 (59.5–171.75)	0.088
PT level, median (IQR)	14.10 (11.40–15.60)	11.70 (10.76–13.65)	0.027
INR, median (IQR)	1.22 (0.99–1.36)	1.01 (0.94–1.18)	0.027
APTT, median (IQR)	31.10 (27.90–37.00)	30.25 (26.35–34.42)	0.438
D-D, median (IQR)	0.38 (0.25–1.41)	0.23 (0.16–0.67)	0.395
TT, median (IQR)	15.2 (13.30–16.80)	14.90 (13.68–15.80)	0.740
ALB, median (IQR)	31.20 (29.50–34.60)	36.50 (33.08–39.05)	0.010
CRP, median (IQR)	67.85 (23.00–94.52)	29.63 (7.31–40.96)	0.184
IL-6, median (IQR)	40.82 (21.22–104.07)	13.28 (2.57–22.03)	0.031
PCT, median (IQR)	1.15 (0.31–4.02)	0.10 (0.07–0.19)	<0.001

### Analysis of long COVID

Among surviving patients with adequate follow-up, seven were classified as long COVID, and 25 as the non-long-COVID survivor group. No significant differences were observed between these two groups with respect to age, gender, or initial COVID-19 severity in this cohort. Univariate analysis identified ICU admission and antiviral treatment as significantly associated with long COVID development, although these findings should be interpreted cautiously given the limited sample size. Furthermore, no significant differences were observed in acute-phase neutrophil, lymphocyte, or platelet counts, coagulation parameters, or inflammatory markers (e.g., CRP, IL-6, PCT) between the surviving COVID-19 patients with and without long COVID (Table 3).

**TABLE 3:** Clinical features and laboratory tests of HM patients with/without long COVID-19 in the disease onset and recovery stages

	Long (*N* = 7) *N* (%)		Not-long (*N* = 25) *N* (%)		*P*-value	
	Onset	Recovery	Onset	Recovery	*P^a^*	*P^b^*
Gender					0.576	
Male	3 (42.9%)	/^*c*^	12 (48.0%)	/		
Female	4 (57.1%)	/	13 (52.0%)	/		
Age, median (IQR, range)	46.0 (33.0–69.0, 21–72)	/	57.0 (37.5–64.0, 23–74)	/	0.767	
Diagnosis					0.161	
Acute leukemia	2 (28.6%)		12 (48.0%)			
Lymphoma	2 (28.6%)		6 (24.0%)			
Myelodysplastic syndromes	1 (14.3%)		1 (4.0%)			
Multiple myeloma	0 (0.0%)		5 (20.0%)			
Others	2 (28.6%)		1 (4.0%)			
Stable disease status					0.646	
Yes	1 (14.3%)		7 (28.0%)			
No	6 (85.7%)		18 (72.0%)			
Vaccination status					0.401	
Vaccinated	3 (42.9%)		16 (64.0%)			
Unvaccinated	4 (57.1%)		9 (36.0%)			
Recent chemotherapy					0.212	
Yes	1 (14.3%)		11 (44.0%)			
No	6 (85.7%)		14 (56.0%)			
CD20-directed therapy					1.000	
Yes	1 (14.3%)		4 (16.0%)			
No	6 (85.7%)		21 (84.0%)			
Antiviral therapy					0.007	
Yes	7 (100.0%)		9 (36.0%)			
No	0 (0.0%)		16 (64.0%)			
COVID-19 infection					0.296	
Critical-Severe	2 (28.6%)	/	3 (12.0%)	/		
Not Severe	5 (71.4%)	/	22 (88.0%)	/		
Admitted to ICU	2 (28.6%)	/	0 (0.0%)	/	0.042	
Anti-COVID-19 Drugs	7 (100.0%)	/	9 (36.0%)	/	0.003	
Neutrophils, median (IQR)	2.51 (1.75–5.15)	3.5 (2.98–7.30)	2.1 (0.9–4.56)	3.2 (1.79–5.35)	0.484	0.504
Lymphocytes, median (IQR)	0.45 (0.10–6.79)	1.0 (0.8–9.4)	0.7 (0.3–1.35)	0.90 (0.50–1.0)	0.436	0.171
PLT, median (IQR)	114.5 (81.75–167.0)	167 (108–223)	91 (43.5–187.5)	116 (65.6–193.5)	0.653	0.359
PT level, median (IQR)	11.0 (10.7–12.85)	10.75 (10.38–12.55)	11.8 (10.75–12.55)	11.4 (10.7–12.63)	0.781	0.319
INR, median (IQR)	0.96 (0.94–1.12)	0.94 (0.91–1.09)	1.03 (0.94–1.09)	0.99 (0.93–1.09)	0.802	0.319
APTT, median (IQR)	29.0 (24.9–36.4)	25.5 (6.62–30.9)	30.8 (26.3–33.4)	29.45 (26.75–34.98)	0.889	0.118
D-D, median (IQR)	0.16 (0.09–0.39)	0.10 (0.58–0.20)	0.35 (0.16–0.69)	0.31 (0.16–0.64)	0.140	0.059
TT, median (IQR)	16.8 (14.45–18.7)	15.95 (13.7–17.68)	14.8 (13.75–15.70)	14.85 (14.18–15.78)	0.126	0.355
ALB, median (IQR)	36.5 (30.3–40.25)	43.6 (35.65–47.2)	36.4 (31.5–40.4)	36.8 (32.4–40.8)	0.980	0.055
CRP, median (IQR)	10.05 (2.60–28.64)	1.93 (0.33–18.81)	34.03 (6.54–49.95)	5.36 (2.31–63.11)	0.254	0.133
IL6, median (IQR)	2.59 (1.86–15.93)	5.36 (1.61–)	13.28 (5.59–28.62)	8.30 (2.40–24.50)	0.197	0.378
PCT, median (IQR)	0.07 (0.03–0.10)	0.055 (0.04–)	0.11 (0.68–0.20)	0.095 (0.068–0.52)	0.103	0.164

^*a*^ Represented the comparison of the baseline for HMs with/without long COVID-19 in the onset stage.

^*b*^ Represented the comparison for HMs with/without long COVID-19 in the recovery stage.

^*c*^ '/’ indicates not applicable.

### Routine laboratory parameters versus proteomic profiling

In this cohort, routine laboratory measurements did not clearly separate survivors later classified as long COVID from those without persistent post-acute symptoms. This suggests that traditional clinical methods, focused on a limited set of laboratory parameters, may fail to detect the underlying molecular differences between these groups. In contrast, proteomic profiling captured broader molecular differences across outcome groups, enabling the identification of specific biomarkers and dysregulated pathways associated with long COVID, thereby providing deeper insights into its biological mechanisms. These findings suggest that serum proteomics may provide complementary biological information beyond conventional laboratory markers, although the present data do not support predictive claims and should be interpreted as association-based.

### Quantitative proteomic profiling of serum samples across clinical stages and outcome groups

**Fig 1:**
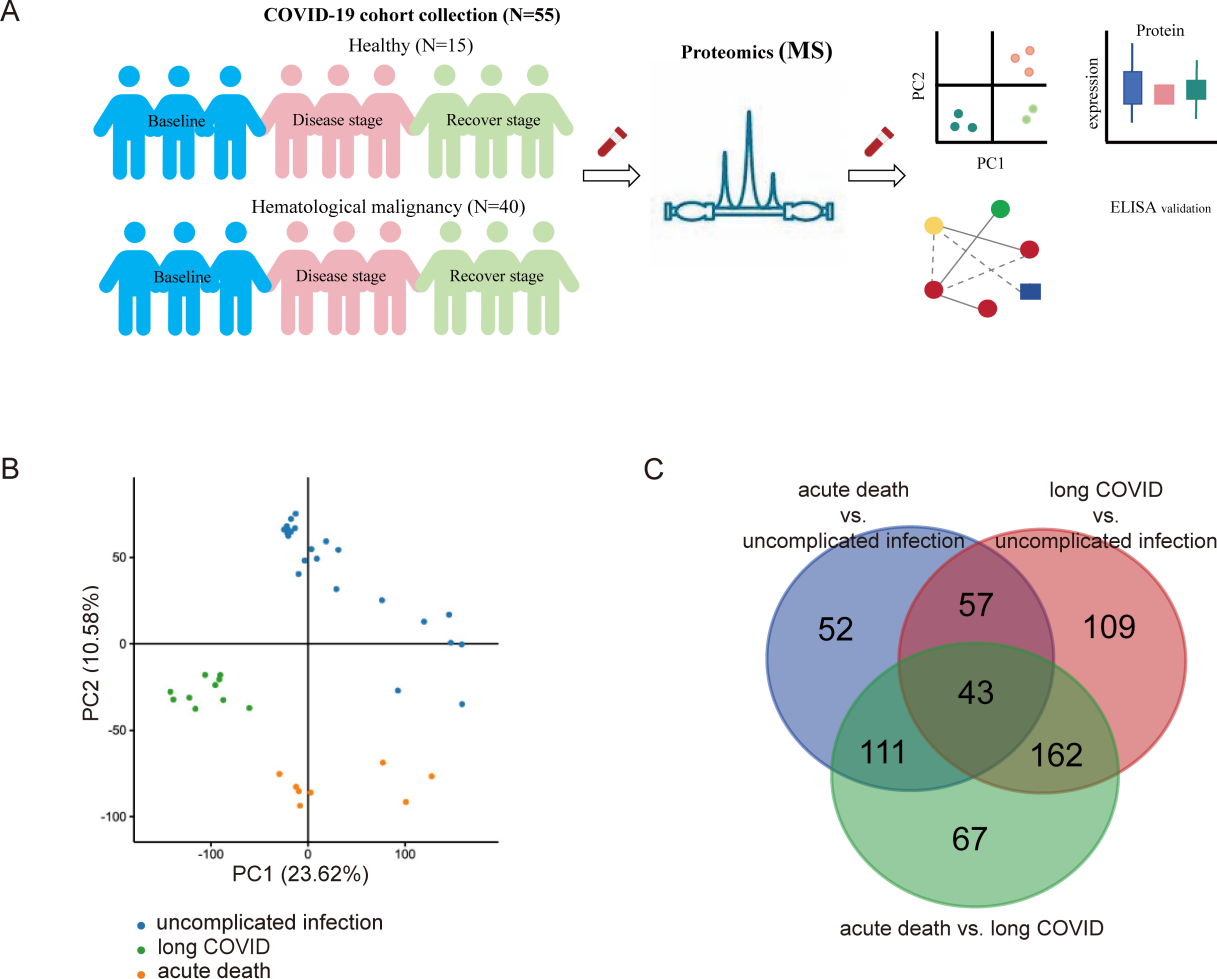
Study design and quantitative serum proteomic profiling in patients with HMs and COVID-19. (**A**) Schematic overview of cohort enrollment, outcome-group assignment, sampling stages, and proteomic workflow. Serum samples were collected from HM patients with COVID-19 and healthy controls. (**B**) Principal component analysis (PCA) showing the distribution of samples across outcome groups. (**C**) Numbers of differentially expressed proteins identified in pairwise comparisons between outcome groups. Differential proteins were defined using effect-size criteria together with FDR-adjusted significance thresholds, as specified in the Materials and Methods and Supplementary Tables.

**Fig 2:**
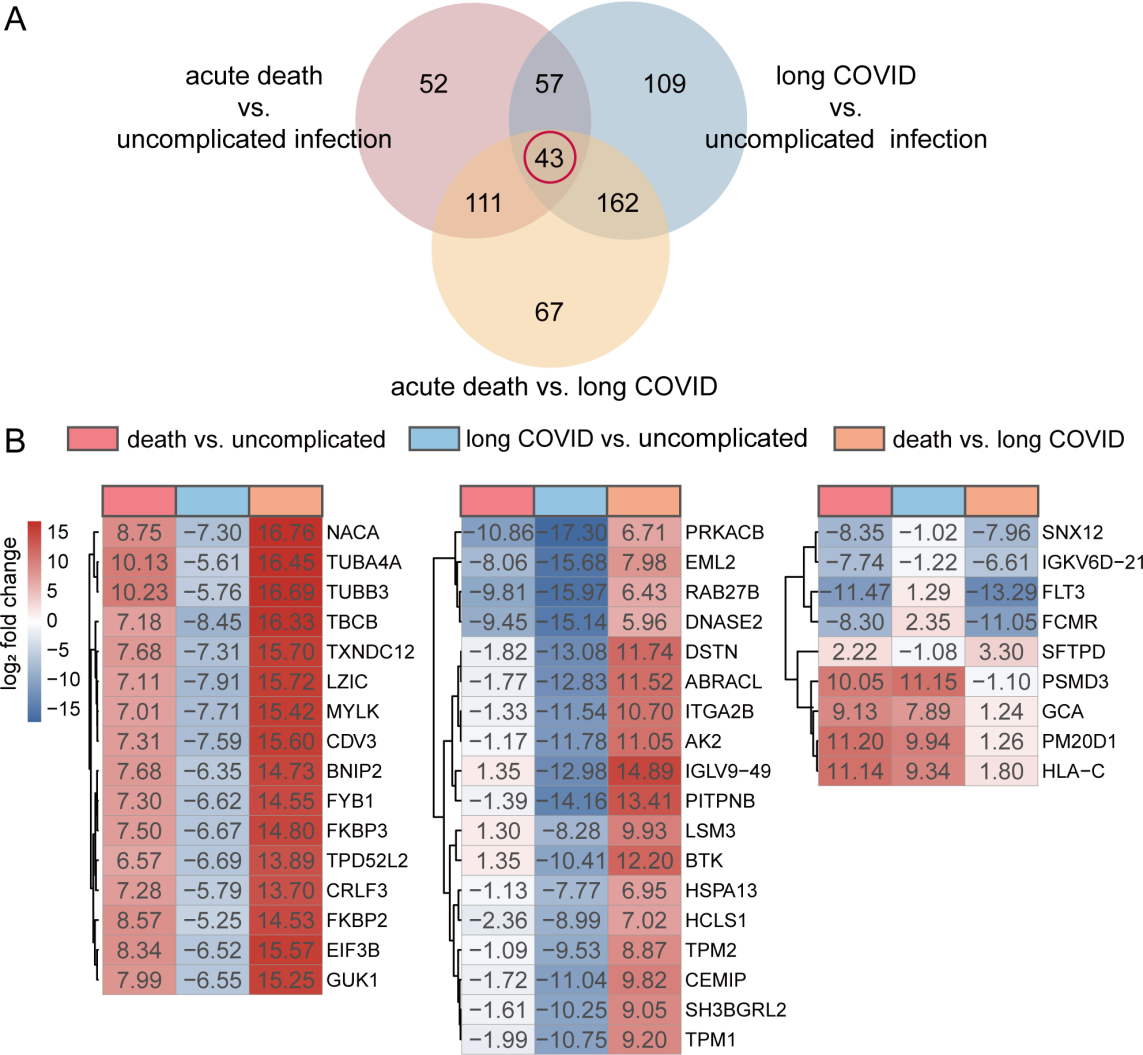
Differentially expressed proteins identified in serum proteomic studies in COVID-19 patients with HM across different clinical outcome groups. (**A**) Venn diagram of differentially expressed proteins between different clinical infection groups. (**B**) Forty-three overlapping proteins of three comparisons between different clinical outcomes.

To investigate proteomic differences associated with distinct clinical trajectories in HM patients with COVID-19, we performed DIA-based quantitative serum proteomics in 40 HM patients and 15 healthy controls. Principal component analysis showed separation among major sample groups (Fig. 1B). A total of 601 proteins were identified across all samples. Pairwise comparisons among the uncomplicated infection, long COVID, and acute death groups identified 263 (acute death group vs uncomplicated infection group), 371 (long COVID group vs uncomplicated infection group), and 383 (acute death group vs long COVID group) significantly differentially expressed proteins (Fig. 1C; Table S1). Across these comparisons, 43 overlapped with disease-stage-specific proteins (Fig. 1C). Numerous proteins were identified as differentially expressed across pairwise comparisons corresponding to different clinical outcomes (Fig. 2; Table S1). These proteins were predominantly involved in the host defense response, innate immune response, and inflammatory signaling. Pathway enrichment analysis highlighted significant involvement of cell-matrix interactions, cytoskeletal remodeling, platelet activation, hypertrophic cardiomyopathy, and dilated cardiomyopathy (Table S2).

### Immune-related protein dysregulation across outcome groups in HM patients with COVID-19

**Fig 3:**
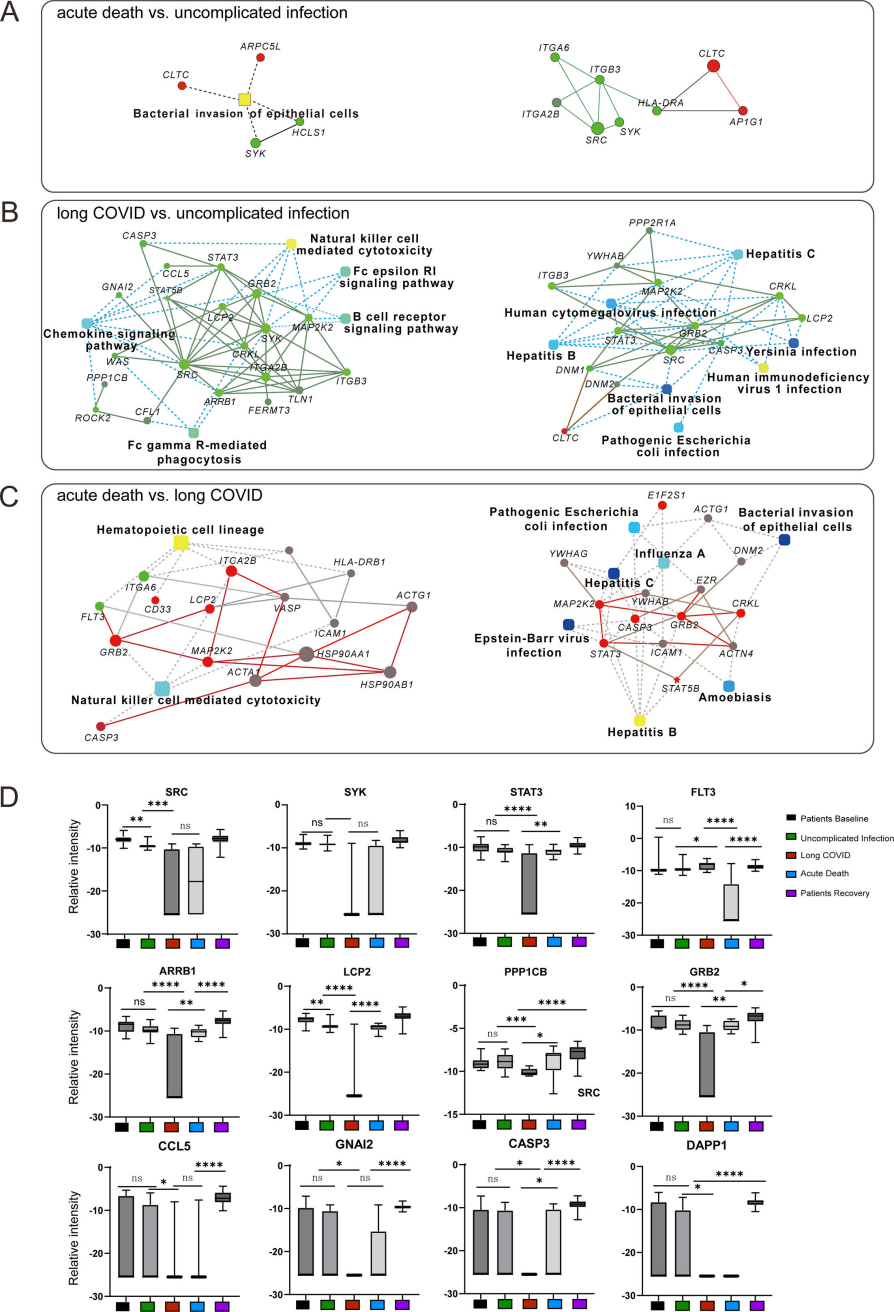
Disruption of pathways related to immune dysregulation in COVID-19 infection with different clinical outcome groups of hematological tumor patients, extending from the baseline stage, disease stage, to the recovery stage. (**A–C**) Enrichment and interaction analyses of dysregulated immune-related pathways in pairwise comparisons between the uncomplicated infection group, long COVID group, and acute death group. Pathway plots include enrichment significance and gene set size. (**D**) Relative abundance of selected proteins across outcome groups. Data are shown as box-and-whisker plots. *q < 0.05, **q < 0.01, ***q < 0.001, ns: not significant.

The interplay between viruses and their hosts involves mutual dependence, competition, and adaptation (17–19). To investigate differences in infection mechanisms among HM patients with different clinical outcomes, we compared acute-phase proteomic profiles among the uncomplicated infection, long COVID, and acute-death groups. KEGG enrichment analysis revealed several pathways related to viral infection processes and antiviral immune responses, including “defense response to virus” and “innate immune response” (Fig. 3; Table S2). Notably, viral entry mediators CLTC (clathrin heavy chain) and AP1G1 (adaptor protein complex 1 gamma 1) were significantly upregulated in fatal cases compared with those with uncomplicated infections (Fig. 3A). Additionally, pathways associated with cell-substrate focal adhesion and cytoskeletal elements, crucial for maintaining endothelial barrier integrity (20), showed marked alterations. Specifically, endothelial integrity markers ITGB3 and ITGA2B showed significant downregulation in the acute death group (21, 22), implicating endothelial dysfunction as a major complication in severe COVID-19 cases (23). These findings suggest that enhanced viral entry and compromised endothelial barrier function contribute to adverse clinical outcomes in COVID-19.

Compared to the uncomplicated infection group, the long COVID group exhibited significant downregulation across multiple antiviral pathways, including antigen processing and presentation, natural killer cell-mediated cytotoxicity, B cell receptor signaling, Fc gamma R-mediated phagocytosis, chemokine signaling, and leukocyte transendothelial migration (Fig. 3B). Pathway activity followed a consistent pattern: uncomplicated infection > acute death > long COVID. Similar suppression was observed in infection-related pathways (bacterial invasion of epithelial cells and viral infections caused by Epstein-Barr virus, cytomegalovirus, and human immunodeficiency virus type 1).

Proteins such as SYK, STAT3, ARRB1, PPP1CB, GRB2, CCL5, GNIA2, CASP3, and DAPP1, which remained stable or slightly decreased in the acute phase of recovery patients, were significantly reduced in patients with long COVID (Fig. 3D), and some of which were also reduced in the acute phase of patients of acute death. These findings suggest a weakened host cellular response in the long-infection and death groups, potentially facilitating pathogen survival and replication, thereby contributing to prolonged infection and disease progression. These findings suggest that differences in the magnitude and pattern of the acute immune response may be associated with subsequent long-term clinical trajectories.

While both long COVID and death groups showed compromised antiviral response, the long-infection group exhibited more pronounced downregulation of key pathways, indicating a severely impaired immune response that may facilitate the pathogen to persist and replicate over an extended duration. In contrast, acute fatality featured rapid immune hyperactivation/dysregulation during the acute phase, driving fatal outcomes.

### Disruption of proteins related to platelet activation and myocardial dysfunction in COVID-19 patients with HM across different clinical outcome groups

Proteins significantly downregulated during the acute phase of uncomplicated infection compared to both the acute death and long COVID groups were enriched in platelet activation and myocardial function pathways. Notably, the acute death group exhibited an overwhelming increase in proteins associated with these pathways, suggesting their association with severe clinical phenotypes.

**Fig 4:**
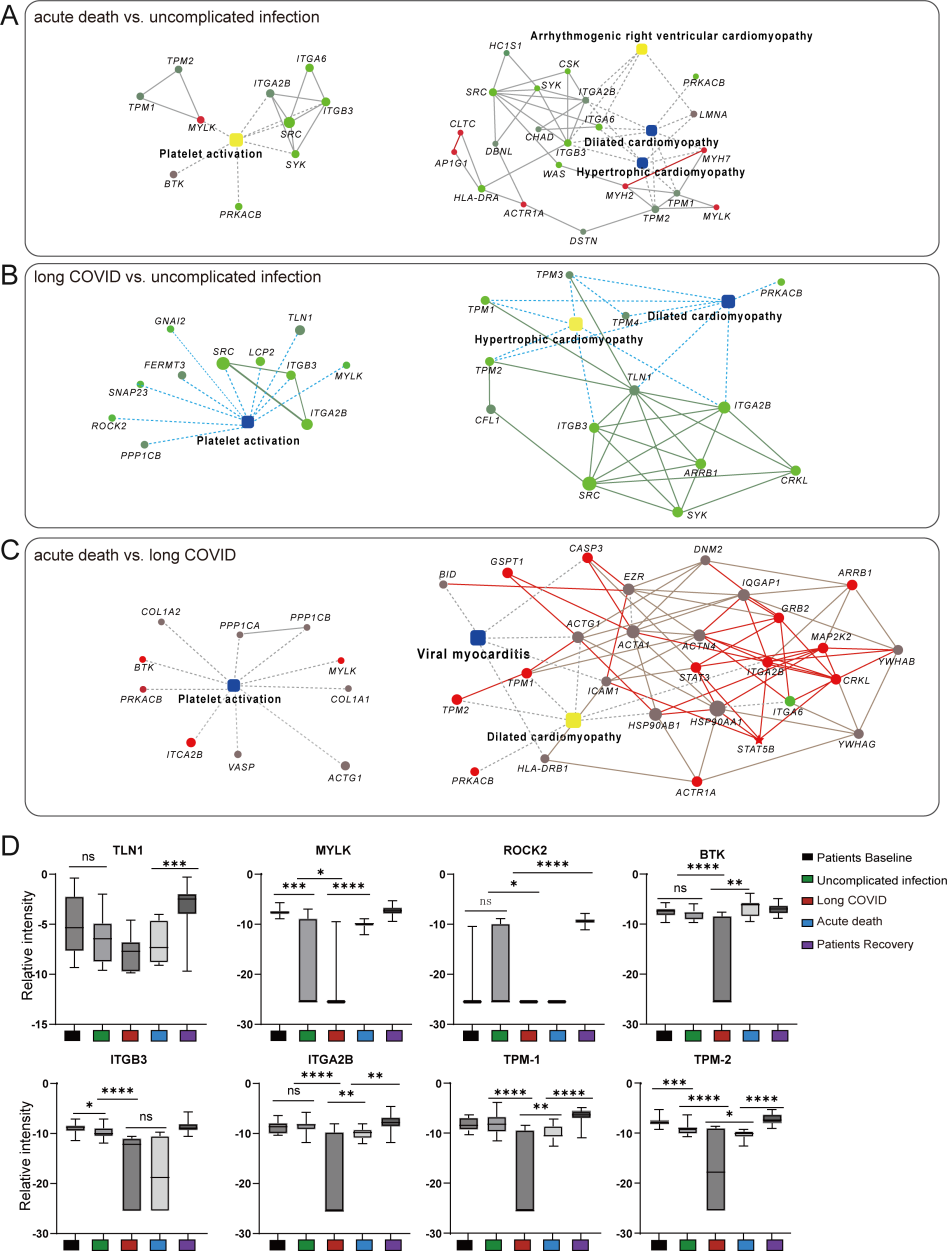
Pathways related to platelet activation and cardiac-related dysfunction across different outcome groups in HM patients with COVID-19. (**A–C**) Interaction analyses of proteins associated with platelet activation and cardiac-related pathways in pairwise comparisons between clinical outcome groups. (**D**) Relative abundance of selected proteins related to platelet activation and cardiac-associated processes. *q < 0.05, **q < 0.01, ***q < 0.001, ns: not significant.

*ITGB3* and *ITGA2B* form the alpha-IIb beta-3 integrin, also known as glycoprotein IIb/IIIa (GP IIb/IIIa), which is predominantly expressed on platelets and plays a critical role in hemostasis and thrombosis (24, 25). Genes associated with integrins and integrin signaling, such as *ITGA6*, *ITGB3*, and *ITGA2B*, were highly enriched. Among these, *ITGA2B* expression was lowest in the long COVID group, followed by the death group, with the uncomplicated infection group exhibiting the highest expression levels (Fig. 4).

Previous studies have suggested that reduced *ITGA2B* expression may indicate disruptions in the regulatory mechanisms of platelet activation and thrombosis (22). Such disruptions may be related to persistent inflammation and symptom burden, although this interpretation remains exploratory in the current cohort. In cases of chronic infection or incomplete viral clearance, *ITGA2B* expression may influence immune cell function and the local microenvironment, exacerbating these symptoms (26). Based on these findings, altered *ITGA2B* expression was associated with clinical outcome groups in this cohort and warrants further evaluation in larger validation studies.

Interestingly, *ITGB3* and *ITGA2B* were also implicated in pathways associated with dilated cardiomyopathy and hypertrophic cardiomyopathy. Proteins involved in these pathways, such as tropomyosin alpha-1 chain (TPM1) and tropomyosin beta chain (TPM2), were significantly upregulated in survivors of uncomplicated infection but were at their lowest levels in the long COVID group. Additional proteins, including TLN1, MYLK, and ROCK2, which are involved in cell migration, cell-to-matrix adhesion, muscle contraction, and actin cytoskeleton dynamics, were also identified (Fig. 4D). Although these pathway annotations do not establish direct cardiac injury in the present cohort, they suggest that cardiac-related biological processes may differ across clinical trajectories. Given prior reports of thrombo-inflammatory and cardiovascular involvement in COVID-19, these observations warrant further evaluation in larger and clinically better-phenotyped cohorts.

**Fig 5:**
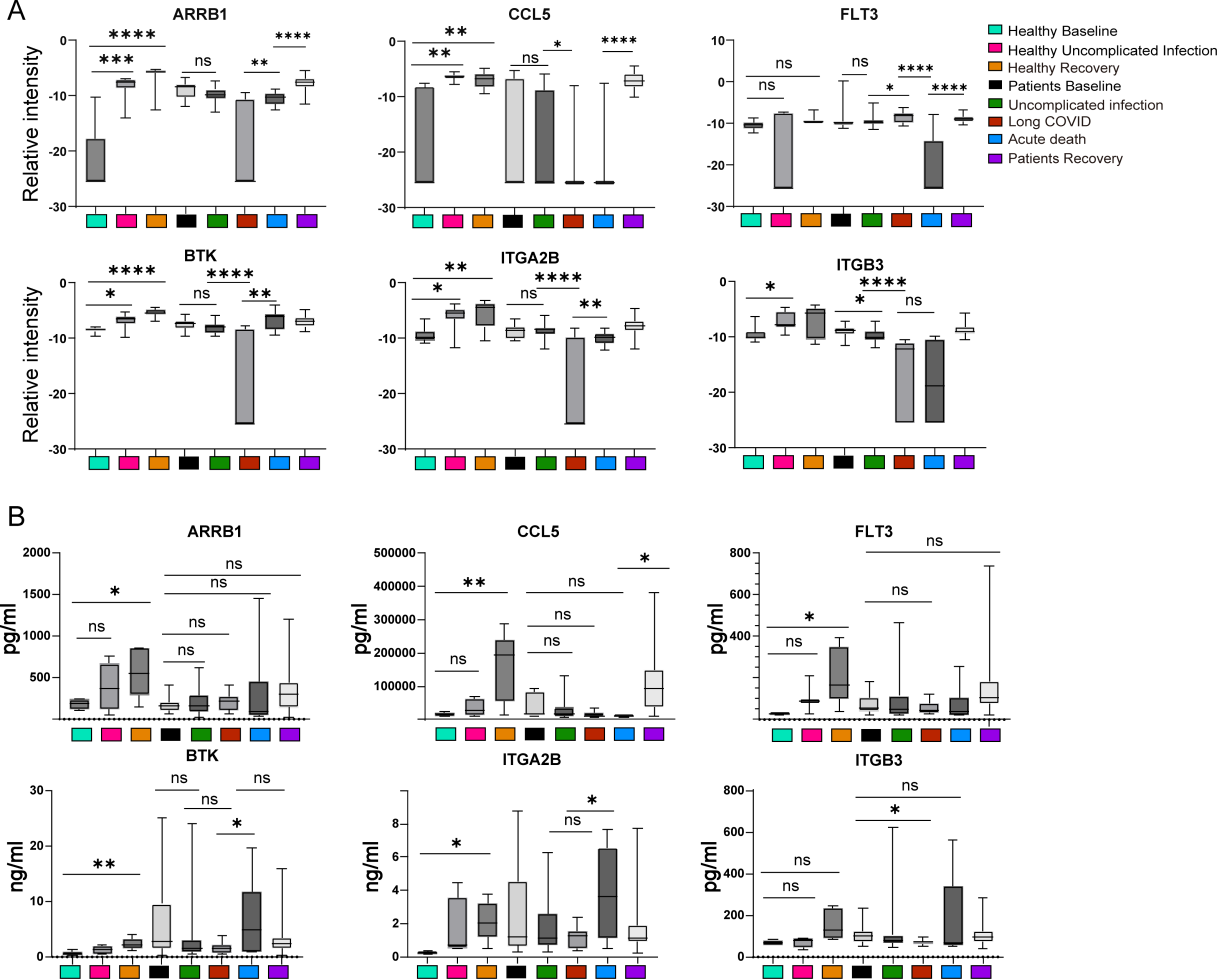
ELISA validation of selected outcome-associated proteins. (**A**) Differential expression patterns of selected proteins identified in proteomic analyses. (**B**) ELISA-based validation of ARRB1, CCL5, FLT3, BTK, ITGA2B, and ITGB3 in serum samples from the indicated groups. *q < 0.05, **q < 0.01, ***q < 0.001, ns: not significant.

To provide orthogonal validation of selected proteomic findings, we performed ELISA assays for ARRB1, CCL5, FLT3, BTK, ITGA2B, and ITGB3. The ELISA results were directionally consistent with the proteomic findings (Fig. 2 and 3) and supported the association of these proteins with outcome-group differences in this cohort (Fig. 5B).

### Network module analysis and drug repositioning

**Fig 6:**
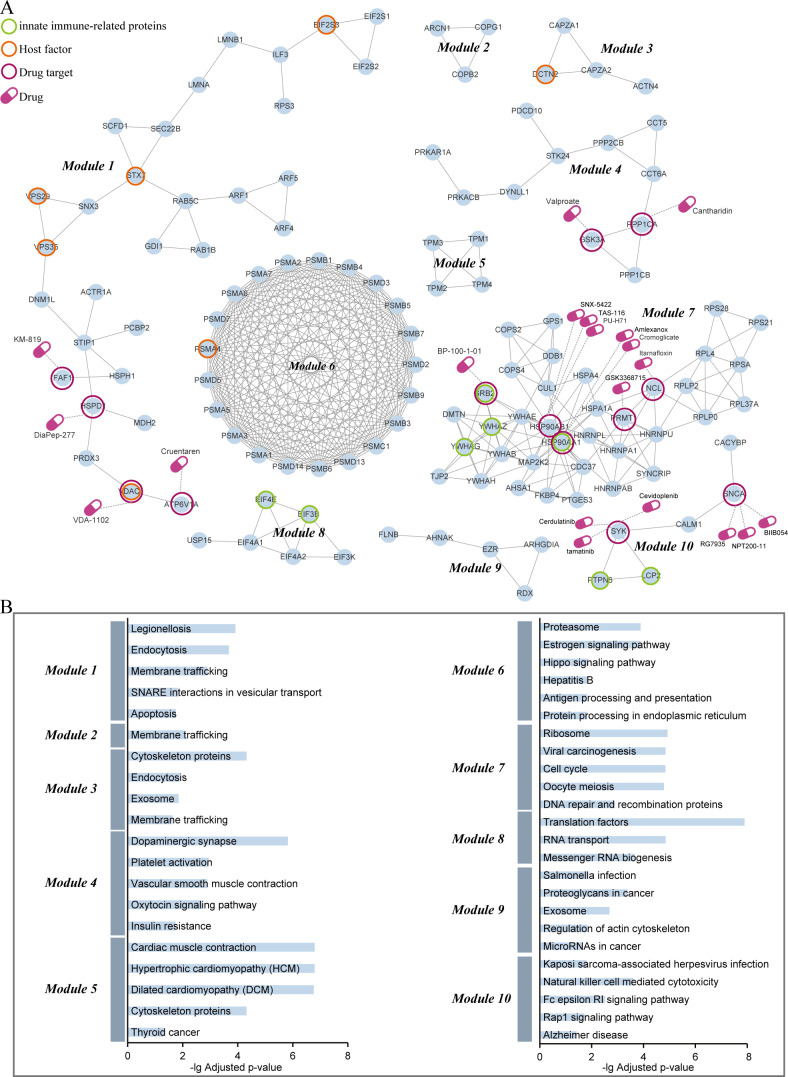
Network module analysis of dysregulated proteins and hypothesis-generating target mapping. (**A**) Interaction network of dysregulated proteins (*n* = 278) and their connections (*n* = 783) in COVID-19 patients (uncomplicated infection, long COVID, acute death groups), clustered into 10 functional modules via Cytoscape MCODE. Nodes are colored by type: orange (immune-related proteins), yellow (host factors), magenta (drug targets), and pink (drugs). Module 6 (central) represents the largest interconnected subnetwork. (**B**) Pathway enrichment analysis of individual modules. These analyses are intended to generate hypotheses regarding potentially relevant pathways and candidate targets for future mechanistic studies.

To further examine the functional relationships among dysregulated proteins across the uncomplicated infection, long-COVID, and acute-death groups, we constructed a protein-protein interaction network based on the combined set of differentially expressed proteins. The resulting network contained 783 interactions among 278 proteins and was further partitioned into 10 modules using MCODE (Fig. 6; Table S3). These modules were enriched in pathways related to immune regulation, membrane trafficking, platelet activation, cardiac muscle contraction, and antigen processing and presentation.

To investigate potential therapeutic opportunities, we performed exploratory drug-target mapping using publicly available databases. This *in silico* analysis identified several proteins with known drug associations, such as SYK (with drugs like tamatinib and cerdulatinib, originally developed for oncology indications), ATP6V1A (linked to cruentaren, an antifungal agent), and SNCA (associated with BIIB054, a Parkinson’s disease therapeutic). Notably, SNCA exhibited a complex expression pattern, being significantly upregulated in acute death patients but downregulated in prolonged infection patients (Fig. 2B), suggesting a potential yet complex role in COVID-19 severity that warrants further study. However, these results should be interpreted strictly as hypothesis-generating, since the present study does not provide functional validation or clinical evidence supporting therapeutic repositioning.

## DISCUSSION

The present study provides a proteomic overview of COVID-19 in patients with hematologic malignancies, a clinically vulnerable population in whom baseline immune dysfunction and treatment-related perturbations may influence host responses to SARS-CoV-2 infection. The emergence of COVID-19 has revealed a spectrum of adverse outcomes, ranging from acute mortality to long COVID, highlighting the intricate interplay between viral factors and host responses (27, 28). Our results suggest that acute-phase proteomic patterns differ according to clinical trajectory. In particular, patients who later developed long COVID showed broader suppression of immune-related pathways, whereas fatal cases showed evidence of dysregulated immune activation during the acute phase. In addition, proteins linked to platelet activation and cardiac-related pathways were associated with adverse outcomes in this cohort.

Importantly, several biological themes identified in this study, including inflammatory dysregulation, thrombo-inflammatory signaling, and cardiac-related pathway perturbation, have also been reported in the broader severe-COVID literature (29–31) and should not be interpreted here as definitively HM-specific. Rather, the value of this study lies in showing how these well-recognized COVID-19-associated processes are represented within a clinically distinct HM population characterized by heterogeneous malignancies, baseline immune impairment, and variable anti-neoplastic treatment exposure. Because this study did not include a non-HM COVID-19 control cohort matched for initial disease severity, ICU admission, and treatment exposure, we cannot determine whether the observed proteomic alterations are specific to hematologic malignancies or instead represent general host responses to severe viral infection and systemic illness.

Our results further suggest that, within the HM setting, adverse trajectories may reflect different balances between impaired antiviral immunity and maladaptive inflammatory responses. The long-COVID group showed broader suppression of immune-related pathways during acute infection, whereas fatal cases showed stronger signals consistent with dysregulated inflammatory activation. These observations are biologically plausible but should be interpreted cautiously because the present study was not designed to establish causality, and the observed proteomic signals may also be influenced by disease severity, ICU admission, treatment exposure, and timing of sampling.

The findings related to platelet activation and cardiac-related pathways are also noteworthy. These pathway-level signals have been implicated previously in severe COVID-19 and post-acute sequelae in non-cancer populations (32–34), and our data suggest that they may also be relevant in HM patients. However, the current study does not support direct mechanistic claims or clinical prediction. Instead, these observations should be viewed as association-based and hypothesis-generating, providing a rationale for future mechanistic work and independent validation in larger cohorts.

This study has several important limitations. First, the cohort was relatively small, especially in the fatal and long-COVID subgroups, which limits statistical power and increases the risk of false-positive findings. Second, this was a single-center study, which may limit generalizability. Third, the HM population was clinically heterogeneous with respect to malignancy subtype, disease status, and treatment exposure, and these factors could not be fully disentangled in the current analysis. Fourth, although repeated sampling was available for a subset of participants, the principal analyses were not designed as formal within-patient longitudinal models, and the temporal structure of the data set therefore remains incomplete. Fifth, despite cautious consideration of key clinical differences across groups, residual confounding cannot be excluded. Finally, because comparator cohorts of non-HM immunocompromised or non-immunocompromised patients were not included, the present study cannot define truly HM-specific proteomic signatures.

In conclusion, our study suggests that distinct acute-phase proteomic patterns are associated with different clinical trajectories in HM patients with COVID-19. These findings extend current understanding of COVID-19 biology in an understudied high-risk population and provide a basis for future mechanistic studies and larger external validation efforts.

## Supplementary Material

Reviewer comments

## Data Availability

The proteomics data set generated in this study has now been deposited in iProX, a ProteomeXchange partner repository, under accession number PXD063172. Further information and requests for resources should be directed to, and will be fulfilled by, the lead contact, Dong Yujun (dongy@hsc.pku.edu.cn).

## SUPPLEMENTAL MATERIAL

Figure S1 spectrum.03996-25-s0001.docx CONSORT flow diagram of study participant recruitment, eligibility, and outcome stratification.

Supplemental tables spectrum.03996-25-s0002.xlsx Tables S1 to S3.

OPEN PEER REVIEW reviewer-comments.pdf An accounting of the reviewer comments and feedback.

ASM does not own the copyrights to Supplemental Material that may be linked to, or accessed through, an article. The authors have granted ASM a non-exclusive, world-wide license to publish the Supplemental Material files. Please contact the corresponding author directly for reuse.
