# Supplementary material for: Proteomic analysis identifies pathways related to immune dysregulation in patients with hematologic malignancies after COVID-19 infection
Source: Microbiol Spectr. 2026 Jul 15;14(8):e03996-25. doi: 10.1128/spectrum.03996-25 (PMC13435943; doi:10.1128/spectrum.03996-25)
Supplement: Supplementary file 1 [file reviewer-comments.pdf]

AMERICAN SOCIETY FOR MICROBIOLOGY | Microbiology Spectrum

Proteomic Analysis identifies Pathways Related to Immune Dysregulation in Patients with Hematologic Malignancies after COVID-19 Infection

Bo Tang, Xiaodi Yang, wenmin Tian, Zhu Jialin, Liu Wei, Di Mingyi, Liu Huihui, Liang Zeyin, Chen Yang, and Dong Yujun

Corresponding Author(s): Bo Tang, Peking University First Hospital

Review Timeline:

Submission Date:	December 10, 2025
Editorial Decision:	February 9, 2026
Revision Received:	May 3, 2026
Editorial Decision:	May 29, 2026
Revision Received:	May 30, 2026
Accepted:	June 2, 2026

Editor: Peter Pelka

Reviewer(s): Disclosure of reviewer identity is with reference to reviewer comments included in decision letter(s). The following individuals involved in review of your submission have agreed to reveal their identity: CLAUDIO Vieira da Silva (Reviewer #2)

Transaction Report:

(Note: With the exception of the correction of typographical or spelling errors that could be a source of ambiguity, letters and reports are not edited. The original formatting of letters and referee reports may not be reflected in this compilation.)

DOI: https://doi.org/10.1128/spectrum.03996-25

Re: Spectrum03996-25 (Proteomic Analysis identifies Pathways Related to Immune Dysregulation in Patients with Hematologic Malignancies after COVID-19 Infection)

Dear Dr. Bo Tang:

Thank you for the privilege of reviewing your work. Below you will find my comments, instructions from the Spectrum editorial office, and the reviewer comments.

Please address the reviewer comments and provide a point-by-point rebuttal to these comments. Also, please ensure the dataset has been deposited to a publicly accessible repository with an accession number.

Please return the manuscript within 60 days; if you cannot complete the modification within this time period, please contact me. If you do not wish to modify the manuscript and prefer to submit it to another journal, notify me immediately so that the manuscript may be formally withdrawn from consideration by Spectrum.

Revision Guidelines
To submit your modified manuscript, log into the submission site at https://spectrum.msubmit.net/cgi-bin/main.plex. Go to Author Tasks and click the appropriate manuscript title to begin. The information you entered when you first submitted the paper will be displayed; update this as necessary. Note the following requirements:

• Upload point-by-point responses to the issues raised by the reviewers in a file named "Response to Reviewers," NOT in your cover letter.
• Upload a compare copy of the manuscript (without figures) as a "Marked-Up Manuscript" file.
• Upload a clean .DOC/.DOCX version of the revised manuscript and remove the previous version.
• Each figure must be uploaded as a separate, editable, high-resolution file (TIFF or EPS preferred), and any multipanel figures must be assembled into one file.
• Any supplemental material intended for posting by ASM should be uploaded with their legends separate from the main manuscript. You can combine all supplemental material into one file (preferred) or split it into a maximum of 10 files with all associated legends included.

For complete guidelines on revision requirements, see our Submission and Review Process webpage. Submission of a paper that does not conform to guidelines may delay acceptance of your manuscript.

Data availability: ASM policy requires that data be available to the public upon online posting of the article, so please verify all links to sequence records, if present, and make sure that each number retrieves the full record of the data. If a new accession number is not linked or a link is broken, provide Spectrum production staff with the correct URL for the record. If the accession numbers for new data are not publicly accessible before the expected online posting of the article, publication may be delayed; please contact production staff (Spectrum@asmusa.org) immediately with the expected release date.

Publication Fees: For information on publication fees and which article types are subject to charges, visit our website. If your manuscript is accepted for publication and any fees apply, you will be contacted separately about payment during the production process; please follow the instructions in that e-mail. Arrangements for payment must be made before your article is published.

ASM Membership: Corresponding authors may join or renew ASM membership to obtain discounts on publication fees. Need to upgrade your membership level? Please contact Customer Service at Service@asmusa.org.

The ASM Journals program strives for constant improvement in our submission and publication process. Please tell us how we can improve your experience by taking this quick Author Survey.

Thank you for submitting your paper to Spectrum.

Sincerely,
Peter Pelka
Editor
Microbiology Spectrum

Reviewer #1 (Comments for the Author):

This manuscript presents a prospective, single-center cohort study of COVID-19 in patients with hematologic malignancies, using DIA-based serum proteomics across disease stages and clinical outcome groups (including death and long COVID/long infection), with ELISA validation of selected proteins. The clinical question is important and the multi-omic approach is potentially

valuable. However, the current version has substantial limitations in cohort definition (particularly long COVID), timing/paired sampling clarity, confounding control (severity and treatment), and statistical rigor for high-dimensional proteomics (multiple testing and modeling of repeated measures). I recommend major revision to improve interpretability and robustness.

Major comments

•The manuscript would benefit from a clearer positioning of what is uniquely contributed beyond existing long-COVID and COVID-severity proteomics literature. Please explicitly state the gap addressed (HM-specific population; longitudinal design; validation) and summarize 2-3 key new biological findings supported by robust statistics.

•Definitions and terminology of long infection vs long COVID is inconsistent and the operational definition of long COVID is not sufficiently specified. Please provide the exact diagnostic criteria for long COVID (time threshold, symptoms, follow-up duration), whether persistent viral positivity is considered (i.e., prolonged infection vs post-acute sequelae), and how missing follow-up was handled. A flow diagram of cohort selection and group assignment is strongly recommended.

•Proteomic profiles are highly time dependent. The manuscript should report, for each group and stage, the time from symptom onset/diagnosis to sampling (median/IQR) and the number of paired samples per participant.

•Severity indicators (e.g., ICU admission) and treatment exposures differ across groups and can independently shift proteomic signals. As written, it is difficult to attribute differences to long COVID biology versus severity/treatment. Please perform multivariable adjustment (age, sex, HM subtype/status, recent chemo, severity/ICU, steroid/antiviral exposure, sampling day), and/or sensitivity analyses restricting to comparable severity strata. Report whether the key proteins/pathways remain significant after adjustment.

•High-dimensional proteomics requires explicit correction for multiple comparisons. Please add a table with FDR-adjusted p-values (q-values) for differential proteins and enrichment analyses. Reliance on nominal $p<0.05$ plus fold-change thresholds is not sufficient, especially with small subgroup sizes.

•If multiple samples per patient are included, analyses should use paired comparisons or mixed-effects models rather than independent-sample tests. Please clarify the statistical approach used for repeated measures and revise accordingly.

•A proteomics pipeline summary (with parameters) would strengthen reproducibility.

•ELISA validation is a strength but needs methodological transparency (sample selection, blinding, replicates, assay CV). If the manuscript suggests predictive utility, this should be supported by validated modeling (cross-validated AUC/calibration). Otherwise, please limit language to associated with and avoid predictive/causal framing.

•Network clustering and drug repositioning should be clearly described (interaction database, confidence thresholds, clustering parameters) and interpreted cautiously as hypothesis-generating. Please avoid therapeutic implications without functional validation.

Minor comments

•PCA and Venn diagrams are descriptive; consider adding ranked tables (supplement) listing key proteins with direction, log2FC, and FDR. For pathway figures, include gene set size and enrichment statistics.

•Standardize use of serum vs plasma throughout.

•For proteomics studies, deposition of raw and processed data is typically expected. Please provide a detailed data availability statement and accession number when available.

•If available, provide vaccination status, variant/time period, and anti-COVID treatment protocols, as these could materially affect outcomes and proteomic signatures.

Reviewer #2 (Comments for the Author):

The study employs mass spectrometry-based proteomics to analyze serum from patients with hematologic malignancies infected with COVID-19. It aims to identify protein signatures that differentiate clinical outcomes: normal infection (recovery), Long COVID, and acute death. Patients under this condition are a high-risk group and are understudied regarding deep proteomic profiling during COVID-19. The use of Data-Independent Acquisition is the current gold standard for biomarker discovery. The data validation is another enhancement to the study. Moreover, the inclusion of drug repositioning strategies adds a valuable translational layer to the research.

To be considered as modifications:

The study includes only 40 patients. Within this cohort, only 8 deaths and 7 Long COVID cases occurred. Such small subgroups severely limit statistical power and increase the risk of false positives , despite the ELISA validation. Recommendation: Authors must explicitly state this as a major limitation and, if possible, expand the cohort.

Hematologic malignancies encompasses vastly different diseases (Acute Leukemia vs. Lymphoma vs. Myeloma). The baseline immune status and treatments (cytotoxic chemotherapy vs. anti-CD20 monoclonal antibodies) vary drastically and influence the serum proteome independently of COVID-19. The manuscript mentions 29 patients had stable disease, but it lacks a detailed analysis of how active oncological treatment impacted protein levels such as ITGA2B (platelets) or cytokines.

The term "normal infection" (line 94) is not technically precise in a medical context. The standard term should be "mild/moderate" or "uncomplicated" infection.

While the definition of Long COVID (4 weeks) follows WHO guidelines, using acute-phase proteomic data to predict an outcome defined 4 weeks later requires a more robust predictive model (e.g., ROC curves), which was not thoroughly detailed.

Many of the identified pathways (platelet activation, myocardial dysfunction, and cytokine storm) are already well-documented in non-cancer patients with severe COVID-19. The paper fails to clearly distinguish what is specific to the "HM + COVID" interaction versus general "severe COVID in immunocompromised patients."

There are typos and non-scientific terms (e.g., "Meyocardium function" in line 46 and section headers.

Summary

This manuscript presents a prospective, single-center cohort study of COVID-19 in patients with hematologic malignancies, using DIA-based serum proteomics across disease stages and clinical outcome groups (including death and long COVID/long infection), with ELISA validation of selected proteins. The clinical question is important and the multi-omic approach is potentially valuable. However, the current version has substantial limitations in cohort definition (particularly long COVID), timing/paired sampling clarity, confounding control (severity and treatment), and statistical rigor for high-dimensional proteomics (multiple testing and modeling of repeated measures). I recommend major revision to improve interpretability and robustness.

Major comments

- The manuscript would benefit from a clearer positioning of what is uniquely contributed beyond existing long-COVID and COVID-severity proteomics literature. Please explicitly state the gap addressed (HM-specific population; longitudinal design; validation) and summarize 2–3 key new biological findings supported by robust statistics.
- Definitions and terminology of long infection vs long COVID is inconsistent and the operational definition of long COVID is not sufficiently specified. Please provide the exact diagnostic criteria for long COVID (time threshold, symptoms, follow-up duration), whether persistent viral positivity is considered (i.e., prolonged infection vs post-acute sequelae), and how missing follow-up was handled. A flow diagram of cohort selection and group assignment is strongly recommended.
- Proteomic profiles are highly time dependent. The manuscript should report, for each group and stage, the time from symptom onset/diagnosis to sampling (median/IQR) and the number of paired samples per participant.
- Severity indicators (e.g., ICU admission) and treatment exposures differ across groups and can independently shift proteomic signals. As written, it is difficult to attribute differences to long COVID biology versus severity/treatment. Please perform multivariable adjustment (age, sex, HM subtype/status, recent chemo, severity/ICU, steroid/antiviral exposure, sampling day), and/or sensitivity analyses restricting to comparable severity strata. Report whether the key proteins/pathways remain significant after adjustment.
- High-dimensional proteomics requires explicit correction for multiple comparisons. Please add a table with FDR-adjusted p-values (q-values) for differential proteins and enrichment analyses. Reliance on nominal $p<0.05$ plus fold-change thresholds is not sufficient, especially with small subgroup sizes.

- If multiple samples per patient are included, analyses should use paired comparisons or mixed-effects models rather than independent-sample tests. Please clarify the statistical approach used for repeated measures and revise accordingly.
- A proteomics pipeline summary (with parameters) would strengthen reproducibility.
- ELISA validation is a strength but needs methodological transparency (sample selection, blinding, replicates, assay CV). If the manuscript suggests predictive utility, this should be supported by validated modeling (cross-validated AUC/calibration). Otherwise, please limit language to associated with and avoid predictive/causal framing.
- Network clustering and drug repositioning should be clearly described (interaction database, confidence thresholds, clustering parameters) and interpreted cautiously as hypothesis-generating. Please avoid therapeutic implications without functional validation.

Minor comments

- PCA and Venn diagrams are descriptive; consider adding ranked tables (supplement) listing key proteins with direction, log2FC, and FDR. For pathway figures, include gene set size and enrichment statistics.
- Standardize use of serum vs plasma throughout.
- For proteomics studies, deposition of raw and processed data is typically expected. Please provide a detailed data availability statement and accession number when available.
- If available, provide vaccination status, variant/time period, and anti-COVID treatment protocols, as these could materially affect outcomes and proteomic signatures.

Response to Reviewers

We sincerely thank the Editor and the reviewers for their thoughtful and constructive comments. These comments have been very helpful in improving the clarity, methodological transparency, and interpretability of our manuscript. In the revised version, we have carefully revised the manuscript to better define the study groups and terminology, clarify the proteomics and ELISA methods, improve the statistical description, strengthen the discussion of confounding and limitations, and moderate statements that could otherwise be interpreted as predictive or causal. We have also deposited the proteomics dataset in a public repository and added the corresponding accession number in the manuscript.

A) Editor / Editorial Office requirements

Comment:

Please ensure the dataset has been deposited to a publicly accessible repository with an accession number.

Response:

We thank the Editor for this important requirement. The proteomics dataset generated in this study has now been deposited in iProX, a ProteomeXchange partner repository, under accession number **PXD063172**. We have added a detailed Data Availability statement to the revised manuscript (**Page 26**).

B) Reviewer #1

Major comment 1

Comment:

The manuscript would benefit from a clearer positioning of what is uniquely contributed beyond existing long-COVID and COVID-severity proteomics literature. Please explicitly state the gap addressed (HM-specific population; longitudinal design; validation) and summarize 2-3 key new biological findings supported by robust statistics.

Response:

We thank the reviewer for this important suggestion. We have revised the Introduction and Discussion to better clarify the specific context and contribution of the present study. In particular,

we now emphasize that our work focuses on patients with hematologic malignancies (HMs), a clinically vulnerable and relatively understudied population with baseline immune dysregulation and heterogeneous treatment exposure, factors that may modify host responses to SARS-CoV-2 infection. We also clarify that the study includes stage-aware sampling and orthogonal ELISA-based validation of selected proteins.

In the revised manuscript, we now summarize the principal findings more explicitly and more cautiously, including: (i) distinct acute-phase proteomic patterns associated with different subsequent clinical trajectories; (ii) broader suppression of immune-related pathways in patients later classified as long COVID and immune hyperactivation in fatal cases; (ii) perturbations in platelet activation- and cardiac-related pathways associated with adverse outcomes; and (iii) associations of platelet activation- and cardiac-related pathways with adverse outcomes, supported by proteomics and ELISA validation of selected proteins.

At the same time, to avoid overstatement, we have moderated the wording throughout and now frame these findings as **association-based and hypothesis-generating**, rather than as definitive HM-specific mechanisms (**Pages 4-5**).

Major comment 2

Comment:

Definitions and terminology of long infection vs long COVID is inconsistent and the operational definition of long COVID is not sufficiently specified. Please provide the exact diagnostic criteria for long COVID (time threshold, symptoms, follow-up duration), whether persistent viral positivity is considered (i.e., prolonged infection vs post-acute sequelae), and how missing follow-up was handled. A flow diagram of cohort selection and group assignment is strongly recommended.

Response:

We agree and have revised the manuscript to improve both terminology and group definition. Throughout the revised version, we have replaced non-standard terms such as “long infection” and “normal infection” with more appropriate terminology, including **“long COVID”** and **“uncomplicated infection”** or **“non-long-COVID survivor group”** as appropriate.

We now define long COVID operationally as the persistence of COVID-19-related symptoms for **≥4 weeks** after the initial SARS-CoV-2 infection, in accordance with the clinical guidance applied during the study period. Persistent viral positivity was **not required** for this classification. We have also clarified the handling of follow-up in the revised Methods and cohort description (**Page 6**). In addition, we have added a participant flow diagram summarizing recruitment, eligibility, sample availability, follow-up, and outcome-group assignment (**Supplementary Figure S1**). Because this was a relatively small single-center cohort, we have also revised the text to present the long-COVID classification cautiously and to avoid implying that it captures the full spectrum of post-acute sequelae (**Page 6**).

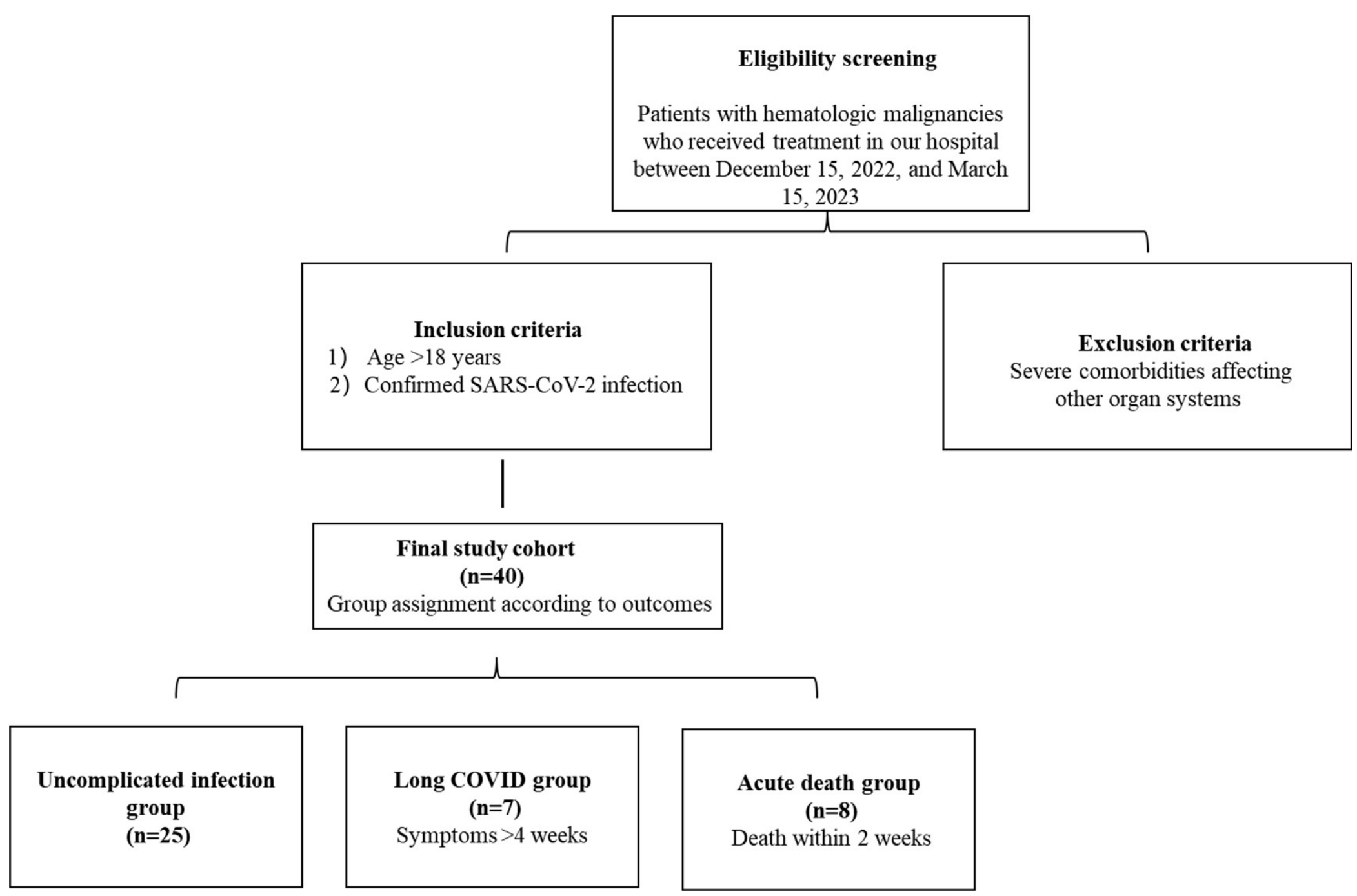

Supplementary Figure S1. CONSORT flow diagram of study participant recruitment, eligibility, and outcome stratification.

Between December 15, 2022, and March 15, 2023, patients with hematologic malignancies treated at our institution were screened for study eligibility. Inclusion criteria were age >18 years and laboratory-confirmed SARS-CoV-2 infection; exclusion criteria were severe comorbidities affecting other organ systems. A total of 40 eligible patients were included in the final cohort and prospectively followed up. Based on their clinical trajectories post-infection, patients were assigned to three groups: (1) uncomplicated infection group (n=25), patients with complete recovery without persistent sequelae;

(2) long COVID group (n=7), patients with symptoms lasting more than 4 weeks after acute infection; (3) acute death group (n=8), patients who died within 2 weeks of SARS-CoV-2 diagnosis.

Major comment 3

Comment:

Proteomic profiles are highly time dependent. The manuscript should report, for each group and stage, the time from symptom onset/diagnosis to sampling (median/IQR) and the number of paired samples per participant.

Response:

We agree and have revised the Methods and Results to provide more explicit information on sampling timing. For each outcome group and sampling stage, we now report the time from diagnosis to blood collection as median (IQR). We also clarify the sample structure at the participant level, including the number of samples contributed per participant and the number of patients with paired acute-phase and recovery samples. This information has been added to improve transparency regarding the time-dependent and longitudinal nature of the proteomic analyses (**Page 6**).

Major comment 4

Comment:

Severity indicators (e.g., ICU admission) and treatment exposures differ across groups and can independently shift proteomic signals. As written, it is difficult to attribute differences to long COVID biology versus severity/treatment. Please perform multivariable adjustment (age, sex, HM subtype/status, recent chemo, severity/ICU, steroid/antiviral exposure, sampling day), and/or sensitivity analyses restricting to comparable severity strata. Report whether the key proteins/pathways remain significant after adjustment.

Response:

We fully agree that disease severity, oncologic background, and treatment exposure are important potential confounders in this cohort. In the revised manuscript, we have expanded the clinical tables and text to provide more complete information on HM subtype, disease status, recent anti-neoplastic therapy, severity/ICU status, vaccination, and anti-COVID

treatment exposure in Table 1, Table 2, and Table 3 (**Pages 40-44**).

Given the limited sample size and the small number of events in the fatal and long-COVID subgroups, we were cautious not to overfit statistical models. Therefore, rather than presenting highly parameterized multivariable models that may be unstable in this cohort, we adopted a parsimonious and descriptive approach, focusing on transparency of clinical differences, cautious interpretation of the proteomic findings, and explicit acknowledgement of residual confounding. Where feasible, we incorporated limited adjusted or stratified analyses and revised the Discussion accordingly.

Importantly, we have tempered the interpretation throughout the manuscript and now explicitly state that the observed proteomic differences cannot be attributed solely to long-COVID biology independent of severity or treatment exposure. We regard these findings as biologically informative but still preliminary, and we now emphasize that larger, better-powered cohorts will be required for more definitive adjusted analyses.

Major comment 5

Comment:

High-dimensional proteomics requires explicit correction for multiple comparisons. Please add a table with FDR-adjusted p-values (q-values) for differential proteins and enrichment analyses. Reliance on nominal $p<0.05$ plus fold-change thresholds is not sufficient, especially with small subgroup sizes.

Response:

Thank you for the suggestion. In the revised manuscript, we have clarified that multiple-testing correction was applied using the Benjamini–Hochberg false discovery rate (FDR) approach. We now report the corresponding adjusted p-values (q-values) in **Supplementary Table S1-S3** for differential protein analysis and pathway enrichment analysis (**Pages 11-12**).

Major comment 6

Comment:

If multiple samples per patient are included, analyses should use paired comparisons or mixed-effects models rather than independent-sample tests. Please clarify the statistical

approach used for repeated measures and revise accordingly.

Response:

We thank the reviewer for raising this point. We have revised the Statistical Analysis section to clarify the analytical framework more explicitly. Although a subset of patients contributed paired acute- and convalescent-phase samples, the principal comparisons in the present study were conducted as stage-matched between-group analyses, using one sample per patient per stage in order to avoid pseudo-replication. The study was therefore not designed as a formal within-patient longitudinal mixed-effects analysis.

We now state this more clearly in the revised manuscript and have also acknowledged in the Discussion that, although repeated sampling was available for many patients, the longitudinal structure of the cohort was incomplete and exploratory.(**Page 13**).

Major comment 7

Comment:

A proteomics pipeline summary (with parameters) would strengthen reproducibility.

Response:

We appreciate this suggestion and have expanded the Methods to provide a more detailed description of the proteomics workflow. Specifically, the revised manuscript now includes additional information on sample preparation, spectral library generation, DIA processing, search database, key search parameters, modification settings, enzyme rules, and FDR control (**Pages 7-11**). We also added a dedicated new subsection titled "Differential Expression Analysis" (**Page 11**) within the Methods. This section describes the data preprocessing steps (imputation, normalization), the statistical tests applied, and the specific thresholds used for defining differentially expressed proteins.

Major comment 8

Comment:

ELISA validation is a strength but needs methodological transparency (sample selection, blinding, replicates, assay CV). If the manuscript suggests predictive utility, this should be supported by validated modeling (cross-validated AUC/calibration). Otherwise, please limit

language to associated with and avoid predictive/causal framing.

Response:

We thank the reviewer for this important suggestion. We have expanded the ELISA Methods to provide greater methodological transparency, including sample selection, blinding procedures, replicate design, and assay reproducibility. Specifically, ELISA assays were performed with technical replicates, and the revised Methods now report assay variability metrics (**Page 12**). At the same time, we fully agree that our study design does not support validated predictive modeling. Therefore, we have removed predictive and causal wording throughout the manuscript and now describe these ELISA-validated proteins as **associated with** clinical outcome groups rather than as validated predictors. This change was necessary because the previous version of the manuscript still contained predictive wording in the Abstract and Introduction (**Page 20**).

Major comment 9

Comment:

Network clustering and drug repositioning should be clearly described (interaction database, confidence thresholds, clustering parameters) and interpreted cautiously as hypothesis-generating. Please avoid therapeutic implications without functional validation.

Response:

Thank you for this constructive suggestion. We have added a dedicated section titled "Network Analysis and Drug Repositioning" in the Methods to describe the network-analysis workflow more clearly, including the source of protein–protein interactions, removal of duplicate/non-physical associations, module detection using MCODE, and downstream pathway/drug-target mapping. We have also revised the Results and Discussion to make the interpretation more cautious.

In the revised manuscript, we now explicitly state that the network and repositioning analyses are **hypothesis-generating** and that the identified candidates should not be interpreted as validated therapeutic options in the absence of functional and clinical validation.

These changes are included in the revised manuscript on **page 12 (Methods).**

Minor comment 10

Comment:

PCA and Venn diagrams are descriptive; consider adding ranked tables (supplement) listing key proteins with direction, log2FC, and FDR. For pathway figures, include gene set size and enrichment statistics.

Response:

Thank you for the suggestion. In response, we have added a new **Supplementary Table S1** that lists the key differentially expressed proteins for all three outcome comparisons. The table includes the direction of change, log2 fold-change, and FDR-adjusted p-value (q-value) for each protein.

Regarding the pathway analysis, the requested gene set size and enrichment statistics for all significant pathways are now provided in **Supplementary Table S2 and Table S3**.

We believe these additions make the statistical support for our findings more transparent and complete.

Minor comment 11

Comment:

Standardize use of serum vs plasma throughout.

Response:

We agree and have standardized the terminology throughout the revised manuscript to **serum**, consistent with the biological samples used for proteomic analysis and ELISA validation.

Minor comment 12

Comment:

For proteomics studies, deposition of raw and processed data is typically expected. Please provide a detailed data availability statement and accession number when available.

Response:

As suggested, a detailed Data Availability statement has now been added, and the raw and processed proteomics data have been deposited in iProX/ProteomeXchange under accession number **PXD063172** (**Page 26**).

Minor comment 13

Comment:

If available, provide vaccination status, variant/time period, and anti-COVID treatment protocols, as these could materially affect outcomes and proteomic signatures.

Response:

Thank you for your suggestion. In the revised manuscript, we have added available clinical context, including vaccination status, study period, and anti-COVID treatment information. Because of the limited sample size, these variables are interpreted cautiously and discussed primarily as potentially relevant contextual factors rather than definitive independent determinants of proteomic differences (**Pages 40-44**).

Reviewer #2

Comment 1

Comment:

The study includes only 40 patients. Within this cohort, only 8 deaths and 7 Long COVID cases occurred. Such small subgroups severely limit statistical power and increase the risk of false positives, despite the ELISA validation. Recommendation: Authors must explicitly state this as a major limitation and, if possible, expand the cohort.

Response:

We thank the reviewer for highlighting this important limitation. We fully agree that the relatively small overall cohort, and particularly the small fatal and long-COVID subgroups, limits statistical power and increases the risk of false-positive findings. We have now made this point more explicit in the Discussion and have moderated the interpretation throughout the manuscript accordingly.

At present, expansion of the cohort is constrained by the number of eligible HM patients treated at our center during the study period. We therefore now explicitly state that larger multicenter validation cohorts will be necessary to confirm the present findings (**Pages 24-25**).

Comment 2

Comment:

Hematologic malignancies encompasses vastly different diseases (Acute Leukemia vs. Lymphoma vs. Myeloma). The baseline immune status and treatments (cytotoxic chemotherapy vs. anti-CD20 monoclonal antibodies) vary drastically and influence the serum proteome independently of COVID-19. The manuscript mentions 29 patients had stable disease, but it lacks a detailed analysis of how active oncological treatment impacted protein levels such as ITGA2B (platelets) or cytokines.

Response:

We agree that the HM cohort is biologically and clinically heterogeneous, and that malignancy subtype, disease status, and treatment exposure may independently influence the serum proteome. In the revised manuscript, we have added more complete descriptive information on HM subtype, disease status, recent chemotherapy, CD20-targeted therapy, and other treatment-related variables. Because of the limited sample size, we were cautious not to over-interpret treatment-specific subgroup analyses. Instead, we now discuss these factors more explicitly as important potential confounders and interpret the observed protein-level differences with greater caution. We also acknowledge in the Discussion that treatment- and subtype-specific effects cannot be reliably disentangled in the current cohort and will require larger, more stratified studies **(Pages 24-25**).

Comment 3

Comment:

The term "normal infection" is not technically precise in a medical context. The standard term should be "mild/moderate" or "uncomplicated" infection.

Response:

We have revised the manuscript to replace "normal infection" with more appropriate terminology, i.e., **"uncomplicated infection"** where this reflects the intended group definition.

Comment 4

Comment:

While the definition of Long COVID (4 weeks) follows WHO guidelines, using acute-phase proteomic data to predict an outcome defined 4 weeks later requires a more robust predictive model (e.g., ROC curves), which was not thoroughly detailed.

Response:

We agree. In the revised manuscript, we have removed predictive framing and now describe the acute-phase proteomic findings as **associated with later long-COVID classification**, rather than as validated predictors. We also explicitly state that the present study was not designed or powered to develop or validate a predictive model (**Page 25**).

Comment 5

Comment:

Many of the identified pathways (platelet activation, myocardial dysfunction, and cytokine storm) are already well-documented in non-cancer patients with severe COVID-19. The paper fails to clearly distinguish what is specific to the "HM + COVID" interaction versus general "severe COVID in immunocompromised patients."

Response:

We thank the reviewer for this insightful comment and agree that this distinction should be discussed more carefully. We have revised the Discussion to better separate shared COVID-19 biology from observations that may be particularly relevant to HM patients. Specifically, we now acknowledge that platelet activation, inflammatory dysregulation, and cardiac-related pathway perturbations are not unique to HM and have been described in the broader severe-COVID literature. The added value of our study lies not in claiming complete biological specificity, but in showing how these pathways appear within a clinically distinct HM population characterized by baseline immune dysregulation, treatment-related immune perturbation, and heterogeneous oncologic exposures. We now explicitly frame our findings as indicating that, in HM patients, adverse trajectories may reflect an altered balance between impaired antiviral immunity and maladaptive inflammatory or thrombo-inflammatory responses, rather than entirely HM-exclusive mechanisms. We also emphasize that larger comparative studies including non-HM immunocompromised and non-immunocompromised COVID-19 cohorts will be required to define true HM-specific signatures (**Page 24-25**).

Comment 6

Comment:

There are typos and non-scientific terms (e.g., "Meyocardium function" in line 46 and section headers).

Response:

We agree and have carefully proofread the revised manuscript to correct typos, non-standard expressions, and terminology inconsistencies. Specifically, terms such as “Meyocardium function” have been corrected to “myocardial dysfunction”. We also corrected additional terminology inconsistencies, including “plasma” versus “serum”, “normal infection” versus “uncomplicated infection”, and “long infection” versus “long COVID”.

Re: Spectrum03996-25R1 (Proteomic Analysis identifies Pathways Related to Immune Dysregulation in Patients with Hematologic Malignancies after COVID-19 Infection)

Dear Dr. Bo Tang:

Thank you for the privilege of reviewing your work. Below you will find my comments, instructions from the Spectrum editorial office, and the reviewer comments.

The manuscript almost meets publication criteria, however, reviewer #2 requested a small modification to the abstract highligting limitations of the study. After this modification is complete the manuscript will be suitable for publication. Please revise and provide a comparison document. Reviewer #1 was fully satisfied. Thank you.

Please return the manuscript within 60 days; if you cannot complete the modification within this time period, please contact me. If you do not wish to modify the manuscript and prefer to submit it to another journal, notify me immediately so that the manuscript may be formally withdrawn from consideration by Spectrum.

Revision Guidelines
To submit your modified manuscript, log into the submission site at https://spectrum.msubmit.net/cgi-bin/main.plex. Go to Author Tasks and click the appropriate manuscript title to begin. The information you entered when you first submitted the paper will be displayed; update this as necessary. Note the following requirements:

• Upload point-by-point responses to the issues raised by the reviewers in a file named "Response to Reviewers," NOT in your cover letter.
• Upload a compare copy of the manuscript (without figures) as a "Marked-Up Manuscript" file.
• Upload a clean .DOC/.DOCX version of the revised manuscript and remove the previous version.
• Each figure must be uploaded as a separate, editable, high-resolution file (TIFF or EPS preferred), and any multipanel figures must be assembled into one file.
• Any supplemental material intended for posting by ASM should be uploaded with their legends separate from the main manuscript. You can combine all supplemental material into one file (preferred) or split it into a maximum of 10 files with all associated legends included.

For complete guidelines on revision requirements, see our Submission and Review Process webpage. Submission of a paper that does not conform to guidelines may delay acceptance of your manuscript.

Data availability: ASM policy requires that data be available to the public upon online posting of the article, so please verify all links to sequence records, if present, and make sure that each number retrieves the full record of the data. If a new accession number is not linked or a link is broken, provide Spectrum production staff with the correct URL for the record. If the accession numbers for new data are not publicly accessible before the expected online posting of the article, publication may be delayed; please contact production staff (Spectrum@asmusa.org) immediately with the expected release date.

Publication Fees: For information on publication fees and which article types are subject to charges, visit our website. If your manuscript is accepted for publication and any fees apply, you will be contacted separately about payment during the production process; please follow the instructions in that e-mail. Arrangements for payment must be made before your article is published.

ASM Membership: Corresponding authors may join or renew ASM membership to obtain discounts on publication fees. Need to upgrade your membership level? Please contact Customer Service at Service@asmusa.org.

The ASM Journals program strives for constant improvement in our submission and publication process. Please tell us how we can improve your experience by taking this quick Author Survey.

Thank you for submitting your paper to Spectrum.

Sincerely,
Peter Pelka
Editor
Microbiology Spectrum

Reviewer #2 (Comments for the Author):

Authors have submitted a revised version of the manuscript. The rsponses to the previous round of review were evaluated. The authors modified the terminology (replacing "normal infection" with "uncomplicated infection"), added the criteria for long COVID,

provided the data repository accession number (PXD063172), and included details regarding the proteomics and ELISA methodologies. The application of False Discovery Rate (FDR) correction for multiple testing is noted, as is the removal of predictive and causal language. However, a fundamental methodological issue remains regarding statistical confounding. The authors opted not to perform multivariable adjustments due to the small sample size (N=40 total; 7 in the long COVID group, 8 in the acute death group). Consequently, the proteomic profiles associated with "acute death" are heavily confounded by baseline disease severity (100% of the acute death group had critical/severe disease, compared to a small fraction of the uncomplicated group). While the authors added this as a limitation in the Discussion, the current presentation still risks implying that these proteomic changes are specific distinct trajectories, rather than a general reflection of severe systemic illness and ICU admission. The limitation regarding disease severity must be stated directly in the Abstract. Because no multivariable adjustment was performed, the Abstract must clarify that the acute-phase proteomic patterns associated with fatal cases and long COVID are closely tied to the initial severity of the disease and cannot be isolated as independent trajectory markers. In the Discussion (Page 22, lines 451-458), the text states that the value of the study lies in showing how these processes are represented within the HM population. The authors must add a clear sentence stating that without a non-HM control group with matched COVID-19 severity, it is impossible to determine if any of the observed proteomic changes are specific to hematologic malignancies, or if they are standard viral responses. Figure 1C still uses the term "normal infection" in the Venn diagram. This must be updated to match the new terminology ("uncomplicated infection") established in the text.

Response to Reviewers

We sincerely thank the Editor and the reviewers for their thoughtful and constructive comments. These comments have been very helpful in improving the clarity, methodological transparency, and interpretability of our manuscript. In the revised version, we have carefully revised the manuscript to better define the study groups and terminology, clarify the proteomics and ELISA methods, improve the statistical description, strengthen the discussion of confounding and limitations, and moderate statements that could otherwise be interpreted as predictive or causal. We have also deposited the proteomics dataset in a public repository and added the corresponding accession number in the manuscript.

A) Editor / Editorial Office requirements

Comment:

Please ensure the dataset has been deposited to a publicly accessible repository with an accession number.

Response:

We thank the Editor for this important requirement. The proteomics dataset generated in this study has now been deposited in iProX, a ProteomeXchange partner repository, under accession number **PXD063172**. We have added a detailed Data Availability statement to the revised manuscript (**Page 26**).

B) Reviewer #1

Major comment 1

Comment:

The manuscript would benefit from a clearer positioning of what is uniquely contributed beyond existing long-COVID and COVID-severity proteomics literature. Please explicitly state the gap addressed (HM-specific population; longitudinal design; validation) and summarize 2-3 key new biological findings supported by robust statistics.

Response:

We thank the reviewer for this important suggestion. We have revised the Introduction and Discussion to better clarify the specific context and contribution of the present study. In particular,

we now emphasize that our work focuses on patients with hematologic malignancies (HMs), a clinically vulnerable and relatively understudied population with baseline immune dysregulation and heterogeneous treatment exposure, factors that may modify host responses to SARS-CoV-2 infection. We also clarify that the study includes stage-aware sampling and orthogonal ELISA-based validation of selected proteins.

In the revised manuscript, we now summarize the principal findings more explicitly and more cautiously, including: (i) distinct acute-phase proteomic patterns associated with different subsequent clinical trajectories; (ii) broader suppression of immune-related pathways in patients later classified as long COVID and immune hyperactivation in fatal cases; (ii) perturbations in platelet activation- and cardiac-related pathways associated with adverse outcomes; and (iii) associations of platelet activation- and cardiac-related pathways with adverse outcomes, supported by proteomics and ELISA validation of selected proteins.

At the same time, to avoid overstatement, we have moderated the wording throughout and now frame these findings as **association-based and hypothesis-generating**, rather than as definitive HM-specific mechanisms (**Pages 4-5**).

Major comment 2

Comment:

Definitions and terminology of long infection vs long COVID is inconsistent and the operational definition of long COVID is not sufficiently specified. Please provide the exact diagnostic criteria for long COVID (time threshold, symptoms, follow-up duration), whether persistent viral positivity is considered (i.e., prolonged infection vs post-acute sequelae), and how missing follow-up was handled. A flow diagram of cohort selection and group assignment is strongly recommended.

Response:

We agree and have revised the manuscript to improve both terminology and group definition. Throughout the revised version, we have replaced non-standard terms such as "long infection" and "normal infection" with more appropriate terminology, including **"long COVID"** and **"uncomplicated infection"** or **"non-long-COVID survivor group"** as appropriate.

We now define long COVID operationally as the persistence of COVID-19-related symptoms for **≥4 weeks** after the initial SARS-CoV-2 infection, in accordance with the clinical guidance applied during the study period. Persistent viral positivity was **not required** for this classification. We have also clarified the handling of follow-up in the revised Methods and cohort description (**Page 6**). In addition, we have added a participant flow diagram summarizing recruitment, eligibility, sample availability, follow-up, and outcome-group assignment (**Supplementary Figure S1**). Because this was a relatively small single-center cohort, we have also revised the text to present the long-COVID classification cautiously and to avoid implying that it captures the full spectrum of post-acute sequelae (**Page 6**).

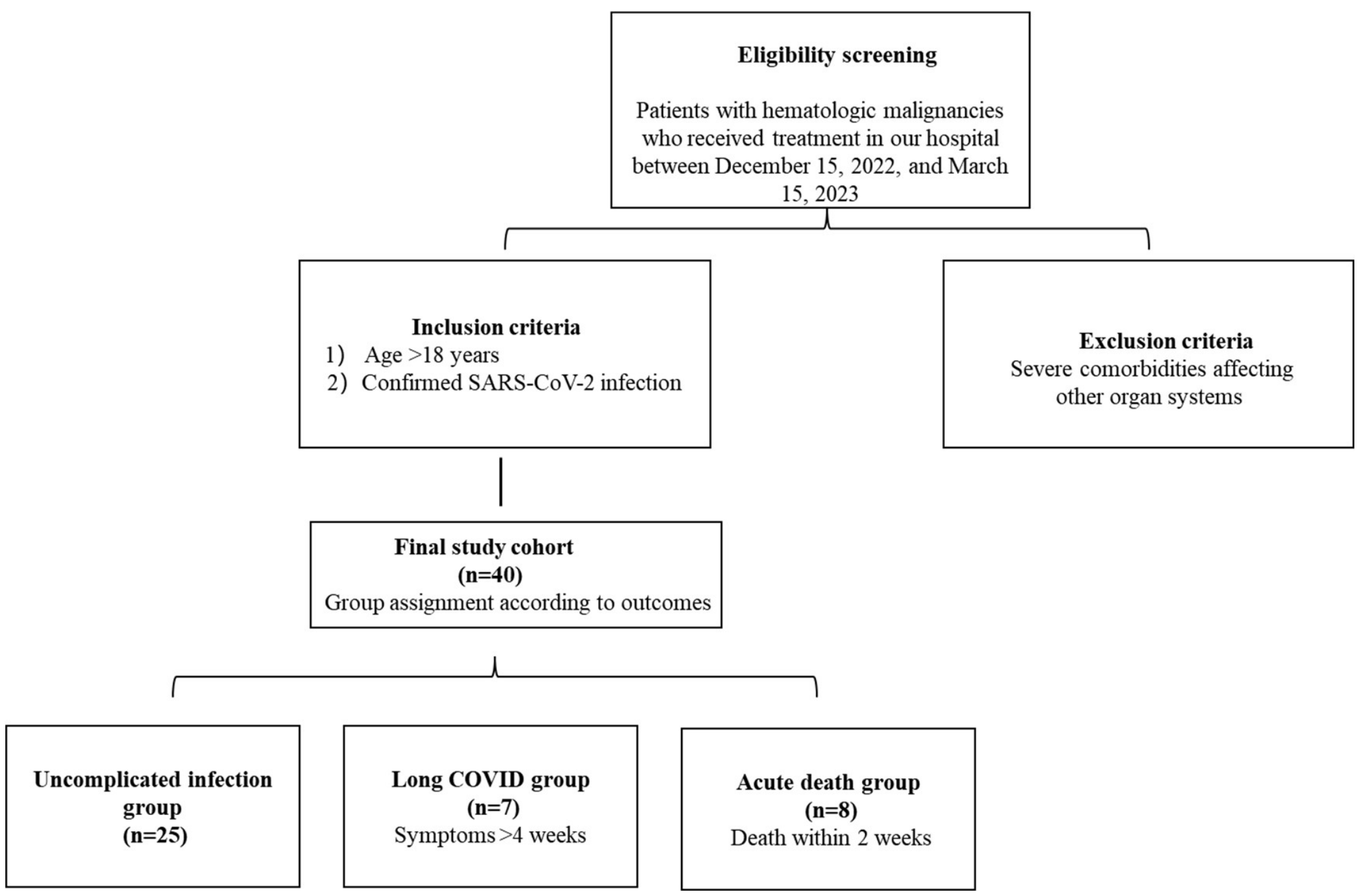

Supplementary Figure S1. CONSORT flow diagram of study participant recruitment, eligibility, and outcome stratification.

Between December 15, 2022, and March 15, 2023, patients with hematologic malignancies treated at our institution were screened for study eligibility. Inclusion criteria were age >18 years and laboratory-confirmed SARS-CoV-2 infection; exclusion criteria were severe comorbidities affecting other organ systems. A total of 40 eligible patients were included in the final cohort and prospectively followed up. Based on their clinical trajectories post-infection, patients were assigned to three groups: (1) uncomplicated infection group (n=25), patients with complete recovery without persistent sequelae;

(2) long COVID group (n=7), patients with symptoms lasting more than 4 weeks after acute infection; (3) acute death group (n=8), patients who died within 2 weeks of SARS-CoV-2 diagnosis.

Major comment 3

Comment:

Proteomic profiles are highly time dependent. The manuscript should report, for each group and stage, the time from symptom onset/diagnosis to sampling (median/IQR) and the number of paired samples per participant.

Response:

We agree and have revised the Methods and Results to provide more explicit information on sampling timing. For each outcome group and sampling stage, we now report the time from diagnosis to blood collection as median (IQR). We also clarify the sample structure at the participant level, including the number of samples contributed per participant and the number of patients with paired acute-phase and recovery samples. This information has been added to improve transparency regarding the time-dependent and longitudinal nature of the proteomic analyses (**Page 6**).

Major comment 4

Comment:

Severity indicators (e.g., ICU admission) and treatment exposures differ across groups and can independently shift proteomic signals. As written, it is difficult to attribute differences to long COVID biology versus severity/treatment. Please perform multivariable adjustment (age, sex, HM subtype/status, recent chemo, severity/ICU, steroid/antiviral exposure, sampling day), and/or sensitivity analyses restricting to comparable severity strata. Report whether the key proteins/pathways remain significant after adjustment.

Response:

We fully agree that disease severity, oncologic background, and treatment exposure are important potential confounders in this cohort. In the revised manuscript, we have expanded the clinical tables and text to provide more complete information on HM subtype, disease status, recent anti-neoplastic therapy, severity/ICU status, vaccination, and anti-COVID

treatment exposure in Table 1, Table 2, and Table 3 (**Pages 40-44**).

Given the limited sample size and the small number of events in the fatal and long-COVID subgroups, we were cautious not to overfit statistical models. Therefore, rather than presenting highly parameterized multivariable models that may be unstable in this cohort, we adopted a parsimonious and descriptive approach, focusing on transparency of clinical differences, cautious interpretation of the proteomic findings, and explicit acknowledgement of residual confounding. Where feasible, we incorporated limited adjusted or stratified analyses and revised the Discussion accordingly.

Importantly, we have tempered the interpretation throughout the manuscript and now explicitly state that the observed proteomic differences cannot be attributed solely to long-COVID biology independent of severity or treatment exposure. We regard these findings as biologically informative but still preliminary, and we now emphasize that larger, better-powered cohorts will be required for more definitive adjusted analyses.

Major comment 5

Comment:

High-dimensional proteomics requires explicit correction for multiple comparisons. Please add a table with FDR-adjusted p-values (q-values) for differential proteins and enrichment analyses. Reliance on nominal $p<0.05$ plus fold-change thresholds is not sufficient, especially with small subgroup sizes.

Response:

Thank you for the suggestion. In the revised manuscript, we have clarified that multiple-testing correction was applied using the Benjamini–Hochberg false discovery rate (FDR) approach. We now report the corresponding adjusted p-values (q-values) in **Supplementary Table S1-S3** for differential protein analysis and pathway enrichment analysis (**Pages 11-12**).

Major comment 6

Comment:

If multiple samples per patient are included, analyses should use paired comparisons or mixed-effects models rather than independent-sample tests. Please clarify the statistical

approach used for repeated measures and revise accordingly.

Response:

We thank the reviewer for raising this point. We have revised the Statistical Analysis section to clarify the analytical framework more explicitly. Although a subset of patients contributed paired acute- and convalescent-phase samples, the principal comparisons in the present study were conducted as stage-matched between-group analyses, using one sample per patient per stage in order to avoid pseudo-replication. The study was therefore not designed as a formal within-patient longitudinal mixed-effects analysis.

We now state this more clearly in the revised manuscript and have also acknowledged in the Discussion that, although repeated sampling was available for many patients, the longitudinal structure of the cohort was incomplete and exploratory.(**Page 13**).

Major comment 7

Comment:

A proteomics pipeline summary (with parameters) would strengthen reproducibility.

Response:

We appreciate this suggestion and have expanded the Methods to provide a more detailed description of the proteomics workflow. Specifically, the revised manuscript now includes additional information on sample preparation, spectral library generation, DIA processing, search database, key search parameters, modification settings, enzyme rules, and FDR control (**Pages 7-11**). We also added a dedicated new subsection titled "Differential Expression Analysis" (**Page 11**) within the Methods. This section describes the data preprocessing steps (imputation, normalization), the statistical tests applied, and the specific thresholds used for defining differentially expressed proteins.

Major comment 8

Comment:

ELISA validation is a strength but needs methodological transparency (sample selection, blinding, replicates, assay CV). If the manuscript suggests predictive utility, this should be supported by validated modeling (cross-validated AUC/calibration). Otherwise, please limit

language to associated with and avoid predictive/causal framing.

Response:

We thank the reviewer for this important suggestion. We have expanded the ELISA Methods to provide greater methodological transparency, including sample selection, blinding procedures, replicate design, and assay reproducibility. Specifically, ELISA assays were performed with technical replicates, and the revised Methods now report assay variability metrics (**Page 12**). At the same time, we fully agree that our study design does not support validated predictive modeling. Therefore, we have removed predictive and causal wording throughout the manuscript and now describe these ELISA-validated proteins as **associated with** clinical outcome groups rather than as validated predictors. This change was necessary because the previous version of the manuscript still contained predictive wording in the Abstract and Introduction (**Page 20**).

Major comment 9

Comment:

Network clustering and drug repositioning should be clearly described (interaction database, confidence thresholds, clustering parameters) and interpreted cautiously as hypothesis-generating. Please avoid therapeutic implications without functional validation.

Response:

Thank you for this constructive suggestion. We have added a dedicated section titled "Network Analysis and Drug Repositioning" in the Methods to describe the network-analysis workflow more clearly, including the source of protein–protein interactions, removal of duplicate/non-physical associations, module detection using MCODE, and downstream pathway/drug-target mapping. We have also revised the Results and Discussion to make the interpretation more cautious.

In the revised manuscript, we now explicitly state that the network and repositioning analyses are **hypothesis-generating** and that the identified candidates should not be interpreted as validated therapeutic options in the absence of functional and clinical validation.

These changes are included in the revised manuscript on **page 12 (Methods).**

Minor comment 10

Comment:

PCA and Venn diagrams are descriptive; consider adding ranked tables (supplement) listing key proteins with direction, log2FC, and FDR. For pathway figures, include gene set size and enrichment statistics.

Response:

Thank you for the suggestion. In response, we have added a new **Supplementary Table S1** that lists the key differentially expressed proteins for all three outcome comparisons. The table includes the direction of change, log2 fold-change, and FDR-adjusted p-value (q-value) for each protein.

Regarding the pathway analysis, the requested gene set size and enrichment statistics for all significant pathways are now provided in **Supplementary Table S2 and Table S3**.

We believe these additions make the statistical support for our findings more transparent and complete.

Minor comment 11

Comment:

Standardize use of serum vs plasma throughout.

Response:

We agree and have standardized the terminology throughout the revised manuscript to **serum**, consistent with the biological samples used for proteomic analysis and ELISA validation.

Minor comment 12

Comment:

For proteomics studies, deposition of raw and processed data is typically expected. Please provide a detailed data availability statement and accession number when available.

Response:

As suggested, a detailed Data Availability statement has now been added, and the raw and processed proteomics data have been deposited in iProX/ProteomeXchange under accession number **PXD063172** (**Page 26**).

Minor comment 13

Comment:

If available, provide vaccination status, variant/time period, and anti-COVID treatment protocols, as these could materially affect outcomes and proteomic signatures.

Response:

Thank you for your suggestion. In the revised manuscript, we have added available clinical context, including vaccination status, study period, and anti-COVID treatment information. Because of the limited sample size, these variables are interpreted cautiously and discussed primarily as potentially relevant contextual factors rather than definitive independent determinants of proteomic differences (**Pages 40-44**).

Reviewer #2

Comment 1

Comment:

The study includes only 40 patients. Within this cohort, only 8 deaths and 7 Long COVID cases occurred. Such small subgroups severely limit statistical power and increase the risk of false positives, despite the ELISA validation. Recommendation: Authors must explicitly state this as a major limitation and, if possible, expand the cohort.

Response:

We thank the reviewer for highlighting this important limitation. We fully agree that the relatively small overall cohort, and particularly the small fatal and long-COVID subgroups, limits statistical power and increases the risk of false-positive findings. We have now made this point more explicit in the Discussion and have moderated the interpretation throughout the manuscript accordingly.

At present, expansion of the cohort is constrained by the number of eligible HM patients treated at our center during the study period. We therefore now explicitly state that larger multicenter validation cohorts will be necessary to confirm the present findings (**Pages 24-25**).

Comment 2

Comment:

Hematologic malignancies encompasses vastly different diseases (Acute Leukemia vs. Lymphoma vs. Myeloma). The baseline immune status and treatments (cytotoxic chemotherapy vs. anti-CD20 monoclonal antibodies) vary drastically and influence the serum proteome independently of COVID-19. The manuscript mentions 29 patients had stable disease, but it lacks a detailed analysis of how active oncological treatment impacted protein levels such as ITGA2B (platelets) or cytokines.

Response:

We agree that the HM cohort is biologically and clinically heterogeneous, and that malignancy subtype, disease status, and treatment exposure may independently influence the serum proteome. In the revised manuscript, we have added more complete descriptive information on HM subtype, disease status, recent chemotherapy, CD20-targeted therapy, and other treatment-related variables. Because of the limited sample size, we were cautious not to over-interpret treatment-specific subgroup analyses. Instead, we now discuss these factors more explicitly as important potential confounders and interpret the observed protein-level differences with greater caution. We also acknowledge in the Discussion that treatment- and subtype-specific effects cannot be reliably disentangled in the current cohort and will require larger, more stratified studies (**Pages 24-25**).

Comment 3

Comment:

The term "normal infection" is not technically precise in a medical context. The standard term should be "mild/moderate" or "uncomplicated" infection.

Response:

We have revised the manuscript to replace "normal infection" with more appropriate terminology, i.e., **"uncomplicated infection"** where this reflects the intended group definition.

Comment 4

Comment:

While the definition of Long COVID (4 weeks) follows WHO guidelines, using acute-phase proteomic data to predict an outcome defined 4 weeks later requires a more robust predictive model (e.g., ROC curves), which was not thoroughly detailed.

Response:

We agree. In the revised manuscript, we have removed predictive framing and now describe the acute-phase proteomic findings as **associated with later long-COVID classification**, rather than as validated predictors. We also explicitly state that the present study was not designed or powered to develop or validate a predictive model (**Page 25**).

Comment 5

Comment:

Many of the identified pathways (platelet activation, myocardial dysfunction, and cytokine storm) are already well-documented in non-cancer patients with severe COVID-19. The paper fails to clearly distinguish what is specific to the "HM + COVID" interaction versus general "severe COVID in immunocompromised patients."

Response:

We thank the reviewer for this insightful comment and agree that this distinction should be discussed more carefully. We have revised the Discussion to better separate shared COVID-19 biology from observations that may be particularly relevant to HM patients. Specifically, we now acknowledge that platelet activation, inflammatory dysregulation, and cardiac-related pathway perturbations are not unique to HM and have been described in the broader severe-COVID literature. The added value of our study lies not in claiming complete biological specificity, but in showing how these pathways appear within a clinically distinct HM population characterized by baseline immune dysregulation, treatment-related immune perturbation, and heterogeneous oncologic exposures. We now explicitly frame our findings as indicating that, in HM patients, adverse trajectories may reflect an altered balance between impaired antiviral immunity and maladaptive inflammatory or thrombo-inflammatory responses, rather than entirely HM-exclusive mechanisms. We also emphasize that larger comparative studies including non-HM immunocompromised and non-immunocompromised COVID-19 cohorts will be required to define true HM-specific signatures (**Page 24-25**).

Comment 6

Comment:

There are typos and non-scientific terms (e.g., "Meyocardium function" in line 46 and section headers).

Response:

We agree and have carefully proofread the revised manuscript to correct typos, non-standard expressions, and terminology inconsistencies. Specifically, terms such as “Meyocardium function” have been corrected to “myocardial dysfunction”. We also corrected additional terminology inconsistencies, including “plasma” versus “serum”, “normal infection” versus “uncomplicated infection”, and “long infection” versus “long COVID”.

Response to Reviewers

We sincerely thank the Editor and the reviewers for their careful evaluation of our manuscript and for the constructive comments and suggestions. We have carefully revised the manuscript in response to these comments. The changes made in the revised manuscript are described point by point below.n number in the manuscript.

Response to Reviewer

Comment:

Authors have submitted a revised version of the manuscript. The rsponses to the previous round of review were evaluated. The authors modified the terminology (replacing "normal infection" with "uncomplicated infection"), added the criteria for long COVID, provided the data repository accession number (PXD063172), and included details regarding the proteomics and ELISA methodologies. The application of False Discovery Rate (FDR) correction for multiple testing is noted, as is the removal of predictive and causal language. However, a fundamental methodological issue remains regarding statistical confounding. The authors opted not to perform multivariable adjustments due to the small sample size (N=40 total; 7 in the long COVID group, 8 in the acute death group). Consequently, the proteomic profiles associated with "acute death" are heavily confounded by baseline disease severity (100% of the acute death group had critical/severe disease, compared to a small fraction of the uncomplicated group). While the authors added this as a limitation in the Discussion, the current presentation still risks implying that these proteomic changes are specific distinct trajectories, rather than a general reflection of severe systemic illness and ICU admission. The limitation regarding disease severity must be stated directly in the Abstract. Because no multivariable adjustment was performed, the Abstract must clarify that the acute-phase proteomic patterns associated with fatal cases and long COVID are closely tied to the initial severity of the disease and cannot be isolated as independent trajectory markers. In the Discussion (Page 22, lines 451-458), the text states that the value of the study lies in showing how these processes are represented within the HM population. The authors must add a clear sentence stating that without a non-HM control group with matched COVID-19 severity, it is impossible to determine if any of the observed proteomic changes are specific to hematologic malignancies, or if they are standard viral responses. Figure 1C still uses the term

"normal infection" in the Venn diagram. This must be updated to match the new terminology ("uncomplicated infection") established in the text.

Response:

We sincerely thank the reviewer for this careful and important comment. We agree that the small cohort size and the imbalance in baseline COVID-19 severity, particularly in the acute death group, represent a major limitation of the present study. We also agree that, because multivariable adjustment was not performed, the observed acute-phase proteomic differences cannot be interpreted as independent trajectory-specific markers.

In response, we have revised the Abstract to explicitly state that the proteomic patterns associated with fatal cases and long COVID are closely intertwined with initial disease severity, ICU admission, and systemic illness, and therefore cannot be isolated as independent trajectory markers. We have also further toned down the language in the Abstract to emphasize association rather than prediction or causality(**Page 2**).

In the Discussion, we added a clear statement that, in the absence of a non-HM COVID-19 control cohort matched for disease severity, it is not possible to determine whether the observed proteomic alterations are specific to hematologic malignancies or instead reflect general host responses to severe viral infection and systemic illness. This revision directly addresses the reviewer’s concern regarding HM specificity(**Page 24-25**).

Finally, we carefully rechecked Figure 1C in the revised manuscript and the corresponding figure file. The label has been corrected to “uncomplicated infection” throughout Figure 1C. To avoid any possible inconsistency between the embedded manuscript figure and the separately uploaded figure file, we have regenerated and re-uploaded the final version of Figure 1.

Re: Spectrum03996-25R2 (Proteomic Analysis identifies Pathways Related to Immune Dysregulation in Patients with Hematologic Malignancies after COVID-19 Infection)

Dear Dr. Bo Tang:

Your manuscript has been accepted, and I am forwarding it to the ASM production staff for publication. Your paper will first be checked to make sure all elements meet the technical requirements. ASM staff will contact you if anything needs to be revised before copyediting and production can begin. Otherwise, you will be notified when your proofs are ready to be viewed.

Data Availability: ASM policy requires that data be available to the public upon online posting of the article, so please verify all links to sequence records, if present, and make sure that each number retrieves the full record of the data. If a new accession number is not linked or a link is broken, provide production staff with the correct URL for the record. If the accession numbers for new data are not publicly accessible before the expected online posting of the article, publication may be delayed; please contact ASM production staff immediately with the expected release date.

Publication Fees: For information on publication fees and which article types have charges, please visit our website. We have partnered with Copyright Clearance Center (CCC) to collect author charges. If fees apply to your paper, you will receive a message from no-reply@copyright.com with further instructions. For questions related to paying charges through RightsLink, please contact CCC at ASM_Support@copyright.com or toll free at +1-877-622-5543. CCC makes every attempt to respond to all emails within 24 hours.

ASM Membership: Corresponding authors may join or renew ASM membership to obtain discounts on publication fees. Need to upgrade your membership level? Please contact Customer Service at Service@asmusa.org.

Embargo Policy: A press release may be issued as soon as the manuscript is posted on the Spectrum Latest Articles webpage. The corresponding author will receive an email with the subject line "ASM Journals Author Services Notification" when the article is available online.

The ASM Journals program strives for constant improvement in our submission and publication process. Please tell us how we can improve your experience by taking this quick Author Survey.

Thank you for submitting your paper to Spectrum.

Sincerely,
Peter Pelka
Editor
Microbiology Spectrum
