# Supplementary material for: Proteomic analysis identifies pathways related to immune dysregulation in patients with hematologic malignancies after COVID-19 infection
Source: Microbiol Spectr. 2026 Jul 15;14(8):e03996-25. doi: 10.1128/spectrum.03996-25 (PMC13435943; doi:10.1128/spectrum.03996-25)
Supplement: Supplementary file 2 [file spectrum.03996-25-s0001.docx]

Supplementary Figure S1. CONSORT flow diagram of study participant recruitment, eligibility, and outcome stratification.

Between December 15, 2022, and March 15, 2023, patients with hematologic malignancies treated at our institution were screened for study eligibility. Inclusion criteria were age >18 years and laboratory-confirmed SARS-CoV-2 infection; exclusion criteria were severe comorbidities affecting other organ systems. A total of 40 eligible patients were included in the final cohort and prospectively followed up. Based on their clinical trajectories post-infection, patients were assigned to three groups: (1) uncomplicated infection group (n=25), patients with complete recovery without persistent sequelae; (2) long COVID group (n=7), patients with symptoms lasting more than 4 weeks after acute infection; (3) acute death group (n=8), patients who died within 2 weeks of SARS-CoV-2 diagnosis
